# DARPP-32 promotes ERBB3-mediated resistance to molecular targeted therapy in EGFR-mutated lung adenocarcinoma

**DOI:** 10.1038/s41388-021-02028-5

**Published:** 2021-10-21

**Authors:** Sk. Kayum Alam, Yongchang Zhang, Li Wang, Zhu Zhu, Christina E. Hernandez, Yuling Zhou, Nong Yang, Jian Lei, Xiaoyan Chen, Liang Zeng, Mark A. Klein, Luke H. Hoeppner

**Affiliations:** 1 The Hormel Institute, University of Minnesota, Austin, MN USA; 2 Department of Medical Oncology, Lung Cancer and Gastrointestinal Unit, Hunan Cancer Hospital/The Affiliated Cancer Hospital of Xiangya School of Medicine, Central South University, Changsha, China; 3 Department of Pathology, Hunan Cancer Hospital/The Affiliated Cancer Hospital of Xiangya School of Medicine, Central South University, Changsha, China; 4 Hematology/Oncology Section, Minneapolis Veterans Affairs Healthcare System, Minneapolis, MN USA; 5 Division of Hematology, Oncology and Transplantation, University of Minnesota, Minneapolis, MN USA; 6 Masonic Cancer Center, University of Minnesota, Minneapolis, MN USA

**Keywords:** Non-small-cell lung cancer, Mechanisms of disease

## Abstract

Epidermal growth factor receptor (EGFR) tyrosine kinase inhibitor (TKI)-refractory lung adenocarcinoma (LUAD) progression is a major clinical problem. New approaches to predict and prevent acquired resistance to EGFR TKIs are urgently needed. Here, we show that dopamine and cyclic AMP-regulated phosphoprotein, Mr 32000 (DARPP-32) physically recruits ERBB3 (HER3) to EGFR to mediate switching from EGFR homodimers to EGFR:ERBB3 heterodimers to bypass EGFR TKI-mediated inhibition by potentiating ERBB3-dependent activation of oncogenic signaling. In paired LUAD patient-derived specimens before and after EGFR TKI-refractory disease progression, we reveal that DARPP-32 and kinase-activated EGFR and ERBB3 proteins are overexpressed upon acquired resistance. In mice, DARPP-32 ablation sensitizes gefitinib-resistant xenografts to EGFR TKIs, while DARPP-32 overexpression increases gefitinib-refractory LUAD progression in gefitinib-sensitive lung tumors. We introduce a DARPP-32-mediated, ERBB3-dependent mechanism the LUAD cells use to evade EGFR TKI-induced cell death, potentially paving the way for the development of therapies to better combat therapy-refractory LUAD progression.

## Introduction

Lung cancer is the leading cause of cancer deaths in the United States and worldwide [1]. Non-small cell lung cancer (NSCLC) represents 80–85% of lung cancer diagnoses, most of which presents as advanced disease with poor prognoses: 1-year survival rates of ~15% and median overall survival of less than 12 months [2]. Targeted therapies for NSCLC expressing oncogenic driver mutations have improved prognoses. An approximately 30% of advanced lung adenocarcinoma (LUAD) patients with epidermal growth factor receptor (EGFR) mutations [3] benefit from treatment with EGFR tyrosine kinase inhibitors (TKIs) [4, 5]. Unfortunately, most LUAD patients develop resistance to EGFR TKIs and rapid disease progression occurs [6, 7]. The 2019 novel coronavirus (COVID-19) pandemic has worsened the health consequences of lung cancer [8]. Lung cancer patients have a higher incidence of COVID-19 and more severe symptoms than cancer-free individuals [9–11], which amplifies the urgency and need for improved lung cancer therapies, including new approaches to prevent or overcome therapy-refractory cancer progression.

EGFR is a member of the ERBB family of receptor tyrosine kinases that bind extracellular growth factors to mediate intracellular signaling [12]. EGFR regulates cell proliferation, survival, differentiation and migration through activation of several signal transduction pathways, including the phosphatidylinositol-3 kinase (PI3K)–AKT–mammalian target of rapamycin (mTOR) cascade, mitogen-activated protein kinase (MAPK) signaling, phospholipase Cγ (PLCγ)–protein kinase C (PKC) pathway, and Janus kinase 2 (JAK2)–signal transducer and activator of transcription 3 (STAT3) signaling [13, 14]. EGFR was identified as a promising therapeutic molecular target based on its overexpression and correlation with poor prognosis in NSCLC [15]. Consequently, two small molecule inhibitors targeting EGFR, gefitinib (Iressa^®^, 2003), and erlotinib (Tarceva^®^, 2004) received FDA approval as treatment for NSCLC patients who had failed chemotherapy [15]. In all, 10% of NSCLC patients treated with EGFR inhibitor responded, mostly women, non-smokers, East Asians, and patients with adenocarcinomas displaying bronchioloalveolar histology. Molecular studies revealed that responders typically possessed EGFR mutations. In-frame deletions of amino acids 747–750 in exon 19 made up 45% of the EGFR mutations and 40–45% consisted of L858R mutations in exon 21 of EGFR [4, 5]. These activating mutations hyperactivate the kinase activity of EGFR to stimulate oncogenic signaling that promotes tumor cell survival, proliferation, differentiation, and migration [16–18]. EGFR TKIs are the recommended first-line therapy for NSCLC patients positive for an EGFR mutation based on trials with gefitinib, erlotinib, and afatinib showing significant improvements in response rate and progression-free survival compared with first-line chemotherapy [19]. Although EGFR mutation-positive patients respond well to first-line EGFR TKIs, NSCLC inevitably progresses in most patients after 9–12 months [20]. In 40–60% of these patients, an exon 20 T790M mutation occurs in EGFR [21, 22]. Osimertinib, a third-generation EGFR TKI targeting the T790M mutation and the primary activating EGFR mutations, can be used to overcome resistance to the first-generation TKIs. However, only ~60% of patients with T790M mutations respond to osimertinib, and in those responding patients, NSCLC progression typically occurs in less than 10 months [23]. Osimertinib has been recently approved in the United States as a first-line treatment for advanced NSCLC patients with EGFR sensitizing mutations [23, 24]. Osimertinib resistance mechanisms and their heterogeneity have been demonstrated in both preclinical and clinical studies [25], including EGFR C797S mutations and histological/phenotypic transformation [26, 27]. Furthermore, acquired resistance to EGFR TKIs can develop through activation of other oncogenic pathways, such as c-MET amplification, activation of the PI3K/AKT pathway, and EGFR-independent phosphorylation of ERBB [28]. Thus, better treatment options to overcome EGFR TKI resistance are necessary.

We recently have identified the role of dopamine signaling in lung cancer [29–31]. In particular, we have shown that dopamine- and cyclic AMP-regulated phosphoprotein, Mr 32000 (DARPP-32), and its N-terminal truncated isoform named t-DARPP, contribute to lung oncogenesis [29, 31]. Here, we demonstrate that DARPP-32 and t-DARPP proteins promote EGFR TKI-refractory disease progression. DARPP-32, and its transcriptional splice variant t-DARPP, are frequently overexpressed in breast, gastric, thoracic, colon, pancreatic, and other adenocarcinomas, where their aberrant upregulation contributes to oncogenesis through regulation of cellular processes, including proliferation, survival, migration, and angiogenesis [31–39]. DARPP-32 was initially discovered as an effector of dopaminergic neurotransmission and as a substrate of dopamine-activated protein kinase A (PKA) [40]. Phosphorylation at T34 by PKA causes DARPP-32-mediated inhibition of protein phosphatase-1 (PP-1) [41]. DARPP-32 is converted to an inhibitor of PKA upon phosphorylation of its T75 residue by cyclin-dependent kinase 5 (Cdk5) [42]. The ability of DARPP-32 to function as either a kinase or a phosphatase inhibitor enables it to precisely modulate dopaminergic neurotransmission [42, 43]. In the early 2000s, El-Rifai and colleagues discovered that DARPP-32 is frequently amplified and upregulated in gastric cancer [33, 36]. Cloning and sequence assembly revealed that a novel transcriptional splice variant of DARPP-32 is also overexpressed in gastric cancer. The N-terminally truncated isoform of DARPP-32, termed t-DARPP, was found to utilize a unique alternative first exon located within intron 1 of *phosphoprotein phosphatase-1 regulatory subunit 1B (PPP1R1B)*, the gene that transcribes DARPP-32 and t-DARPP proteins [36]. t-DARPP lacks the first 36 amino acids of DARPP-32, including the T34 phosphorylation residue required for DARPP-32-mediated PP-1 inhibition [36]. Elevated expression of t-DARPP isoform in NSCLC is associated with poor overall survival and increasing tumor (T) stage [31]. Our findings presented in this report suggest that overexpression of DARPP-32 isoforms in EGFR-mutated NSCLC promotes EGFR:ERBB3 “bypass signaling” that enables tumor cells to evade EGFR TKI monotherapy-induced apoptosis by potentiating oncogenic AKT and ERK signaling.

In the context of other cancer types, DARPP-32 proteins have been implicated in therapy-refractory tumor progression. In HER2-positive breast cancer, t-DARPP overexpression promotes resistance to lapatinib, potentially by disrupting lapatinib-induced BIM accumulation to prevent apoptosis [35]. In breast and esophageal cancer, t-DARPP promotes cancer cell survival through PI3K/AKT signaling and mediates trastuzumab (Herceptin) resistance [44–46]. In breast cancer cells, t-DARPP activates insulin-like growth factor 1 receptor (IGF-1R) signaling through heterodimerization with EGFR and HER2 to stimulate glycolysis and trastuzumab resistance [47]. Correspondingly, DARPP-32-mediated IGF-1R activation promotes STAT3 signaling in gastric cancer cells [48]. Overexpression of DARPP-32 has been shown to block gefitinib-induced apoptosis in gastric tumors by promoting EGFR and ERBB3 heterodimerization [49]. Our findings presented here suggest DARPP-32 drives gefitinib-refractory lung tumor growth through similar ERBB3-mediated resistance mechanisms.

## Results

### DARPP-32 is upregulated in EGFR TKI-resistant NSCLC cells

**Fig. 1:**
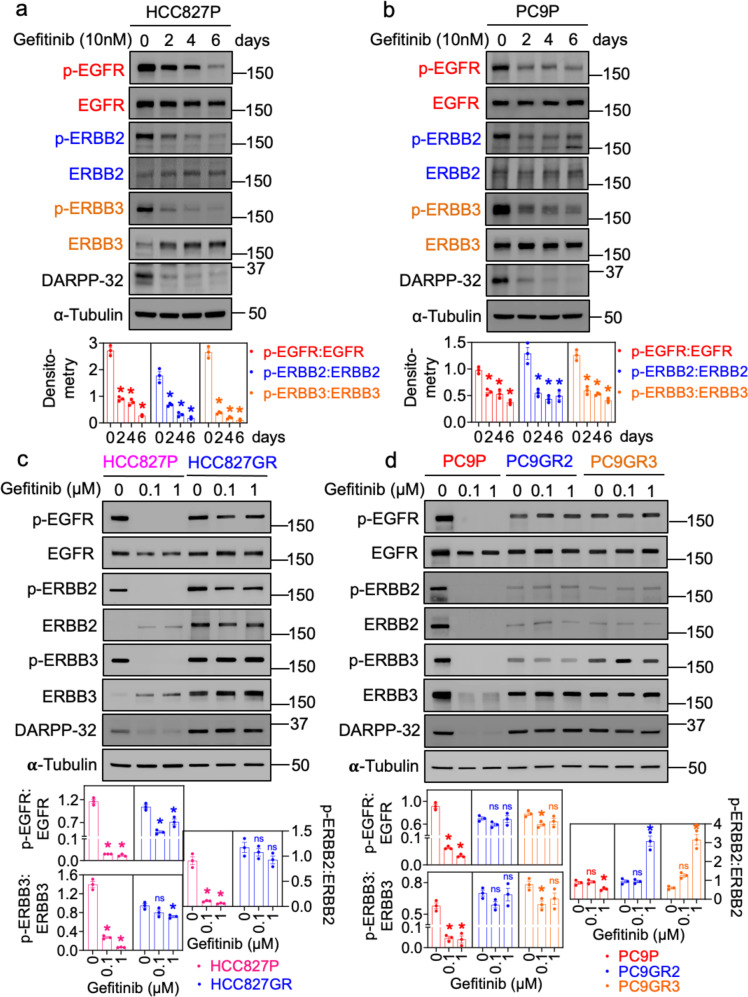
DARPP-32 is upregulated in gefitinib-resistant cells. **a**, **b** Human NSCLC HCC827P (**a**) and PC9P (**b**) cells treated with 10 nM gefitinib at indicated days were lysed and immunoblotted with antibodies against phosphorylated EGFR (p-EGFR), total EGFR (T-EGFR), phosphorylated ERBB2 (p-ERBB2), total ERBB2 (T-ERBB2), phosphorylated ERBB3 (p-ERBB3), total ERBB3 (T-ERBB3), DARPP-32, and α-tubulin (loading control). **c**, **d** HCC827P, HCC827GR (**c**), PC9P, PC9GR2, and PC9GR3 (**d**) cells treated with either 100 nM or 1 µM gefitinib for 24 h were lysed and antibody-reactive protein bands were detected using p-EGFR, EGFR, p-ERBB2, ERBB2, p-ERBB3, ERBB3, DARPP-32, and α-tubulin antibodies. Immunoblotting experiments were replicated independently at least three times, and a representative experimental result is shown. Quantitation of three western blot results performed using ImageJ software has been represented as bar diagrams in the bottom of each figure panel. *P ≤ 0.05, 1-way ANOVA followed by Dunnett’s multiple comparison testing.

Given the ability of DARPP-32 to modulate oncogenic signaling [50, 51], we hypothesized that DARPP-32 contributes to acquired EGFR TKI resistance in NSCLC. To test this hypothesis, we utilized two well-characterized NSCLC models of EGFR TKI resistance. Gefitinib-resistant HCC827 (EGFR^*ΔE746-A750*^) human LUAD cells were previously generated through 6 months of exposure to increasing concentrations of gefitinib and shown to have acquired gefitinib resistance through a c-MET amplification [52]. Secondly, we relied on gefitinib-sensitive PC9 (EGFR^*L858R*^) human LUAD cells and their corresponding PC9 gefitinib-resistant (PC9GR2 and PC9GR3) counterparts, which acquired gefitinib resistance through a secondary EGFR^*T790M*^ mutation following prolonged parental cell exposure to this first-generation EGFR TKI [53]. The half maximal effective concentration (i.e., EC_50_) of gefitinib is 100-fold higher in the resistant cells relative to their parental equivalent (Supplementary Fig. 1a, b). We observed an increase in cell death as well as reduced DARPP-32 protein levels in gefitinib-sensitive, HCC827 parental (HCC827P), and PC9 parental (PC9P) cells upon treatment with EGFR inhibitor, gefitinib (Supplementary Fig. 2, Fig. 1a, b, and Supplementary Fig. 3a, b). Based on this result, we sought to examine DARPP-32 protein levels in gefitinib-resistant EGFR-mutated NSCLC cells. By immunoblotting, we observed elevated DARPP-32 protein expression in gefitinib-resistant cells relative to parental counterparts (Supplementary Fig. 4a, b).

### DARPP-32 overexpression is associated with decreased EGFR TKI-induced NSCLC cell death

**Fig. 2:**
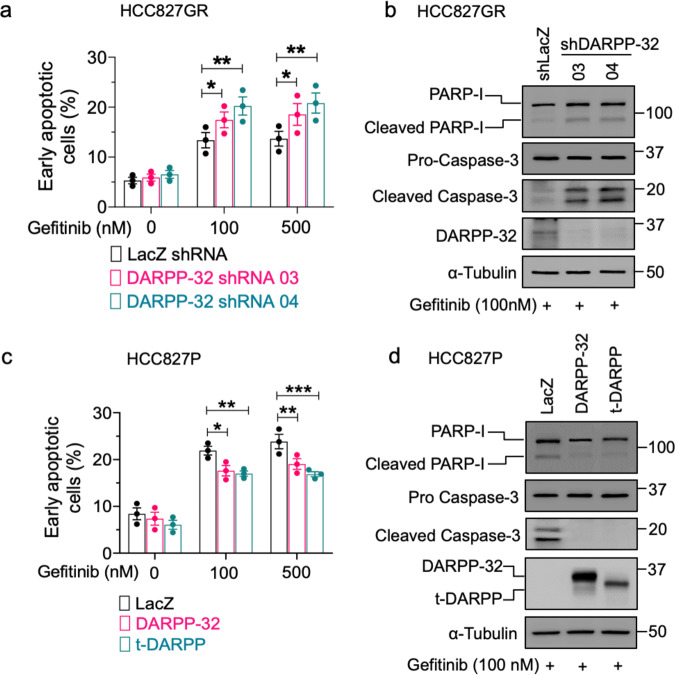
Overexpression of DARPP-32 represses gefitinib-induced cell apoptosis. **a** Human NSCLC HCC827GR cells were transduced with lentivirus encoding control (LacZ) or DARPP-32 shRNAs. Cells treated with increasing doses of gefitinib for 24 h were used to measure apoptosis using FITC-conjugated anti-annexin V antibodies. **b** Immunoblotting was performed using lysates from HCC827GR cells transduced with LacZ or DARPP-32 shRNAs using antibodies against cleaved and uncleaved PARP-I, cleaved and uncleaved (i.e., pro-) caspase-3, DARPP-32 and α-tubulin (loading control). **c** Human NSCLC HCC827P cells were transduced with retrovirus containing control (LacZ), DARPP-32- or t-DARPP-overexpressing clones. Flow cytometry-based apoptosis analysis was performed in gefitinib-treated cells to detect annexin V-positive cells. **d** HCC827P cells overexpressing LacZ, DARPP-32, or t-DARPP were lysed and separated in SDS-PAGE. Immune-reactive protein bands were detected using PARP-I, caspase-3, DARPP-32, and α-tubulin antibodies. Each open circle on a graph represents an independent experiment. Cells were treated with 100 nM gefitinib for 24 h prior to the western blotting experiments. Immunoblot experiments were repeated at least three times. The average number of annexin V-positive cells among three independent experiments were plotted in a bar graph. Error bars indicate standard error of mean (SEM; *n* = 3). *P < 0.05, **P < 0.01, and ***P < 0.001, 2-way ANOVA followed by Dunnett’s test for multiple comparison.

Given that DARPP-32 is upregulated in gefitinib-resistant NSCLC cells, we designed experiments to assess the functional effects of DARPP-32 overexpression in the presence of EGFR TKI. We stably silenced DARPP-32 protein expression in HCC827GR cells via lentiviral-mediated transduction of two previously validated shRNAs [29, 31] (Supplementary Fig. 5a) and then analyzed cell viability in the presence of increasing gefitinib concentrations (Supplementary Fig. 5b). As evident by EC_50_ values, depletion of DARPP-32 decreases cell viability in HCC827GR cells upon gefitinib treatment (Supplementary Fig. 5b, c). We next stably overexpressed DARPP-32 in HCC827P cells (Supplementary Fig. 5d) and measured cell viability upon incubation with increasing concentrations of gefitinib (Supplementary Fig. 5e). HCC827P cells overexpressing DARPP-32 isoforms exhibit a greater EC_50_ value relative to controls (Supplementary Fig. 5e, f), suggesting that overexpression of DARPP-32 promotes resistance to gefitinib. We next sought to understand how DARPP-32 isoforms regulate EGFR-mutated NSCLC cell survival in the presence of gefitinib. We performed flow cytometry-based annexin V apoptosis studies in HCC827GR cells treated with increasing concentrations of gefitinib. We observed increased apoptotic cell death in DARPP-32-depleted HCC827GR cells upon gefitinib treatment compared to LacZ shRNA-transduced control cells (Fig. 2a and Supplementary Fig. 6). We next measured the expression of Poly (ADP-ribose) polymerase 1 (PARP1) and caspase-3 proteins in gefitinib-resistant, DARPP-32-silenced HCC827 and PC9 cells by western blot analysis. PARP-I and caspase-3 produce specific proteolytic cleavage fragments that are well-established surrogates of apoptotic cellular death [54]. We observed an increase in the expression of PARP-I and caspase-3 cleavage fragments in gefitinib-treated, DARPP-32-ablated cells relative to controls (Fig. 2b and Supplementary Fig. 7a, b), suggesting an anti-apoptotic role of DARPP-32. To validate that DARPP-32 overexpression does indeed promote cell survival, we performed annexin V assays. Indeed, our data suggest that stable overexpression of DARPP-32 isoforms protects HCC827P cells from gefitinib-induced apoptosis (Fig. 2c and Supplementary Fig. 8). Correspondingly, we observed a decrease in PARP-I and caspase-3 cleavage, suggesting that overexpression of DARPP-32 isoforms in HCC827P and PC9P cells reduces gefitinib-mediated apoptotic cell death (Fig. 2d and Supplementary Fig. 7c). Collectively, our findings suggest DARPP-32 reduces gefitinib-induced apoptosis of EGFR-mutated NSCLC cells.

### DARPP-32 upregulation and increased ERBB3 activation correlate with EGFR TKI resistance

We next sought to determine the molecular basis of DARPP-32-mediated cell survival in the presence of EGFR inhibition. DARPP-32 has been shown to promote resistance of gastric cancer cells to EGFR inhibitors by promoting an interaction between EGFR and ERBB3, which drives PI3K/AKT signaling [49] to “bypass” EGFR TKI resistance. Importantly, we observe concomitant decreases in DARPP-32 protein expression and phosphorylation of EGFR, ERBB2, and ERBB3 over time when gefitinib-sensitive HCC827P and PC9P cells were treated with various doses of gefitinib (Fig. 1a, b and Supplementary Fig. 3a, b). We next replicated these parental cell experiments relative to their gefitinib-resistant counterparts in the presence of two doses of gefitinib (i.e., 0.1 and 1 µM). The gefitinib-induced decreases in DARPP-32, p-EGFR, and p-ERBB3 that were observed in gefitinib-sensitive HCC827P and PC9P cells do not occur in gefitinib-resistant cells; HCC827GR, PC9GR2, and PC9GR3 all express robust levels of DARPP-32, p-EGFR, and p-ERBB3 in the presence and absence of gefitinib (Fig. 1c, d). Total ERBB2 and ERBB3 protein levels were markedly increased upon EGFR TKI treatment in HCC827 parental cells, and these high levels of ERBB2 and ERBB3 protein expression were maintained in gefitinib-treated HCC827GR cells (Fig. 1c). Moreover, gefitinib-resistant PC9 cells express higher amounts of ERBB2 and ERBB3 proteins in the presence of gefitinib compared to its parental counterpart treated with equal amount of EGFR TKI (Fig. 1d). Phosphorylated and total ERBB4 protein was undetectable in parental and resistant cells (data not shown). Taken together, our observations suggest that upregulation of p-ERBB3, total ERBB3 and total DARPP-32 protein levels positively correlate with an EGFR TKI resistance phenotype in EGFR-mutated NSCLC cells.

### DARPP-32 stimulates ERBB3 activation in EGFR TKI-treated NSCLC cells

**Fig. 3:**
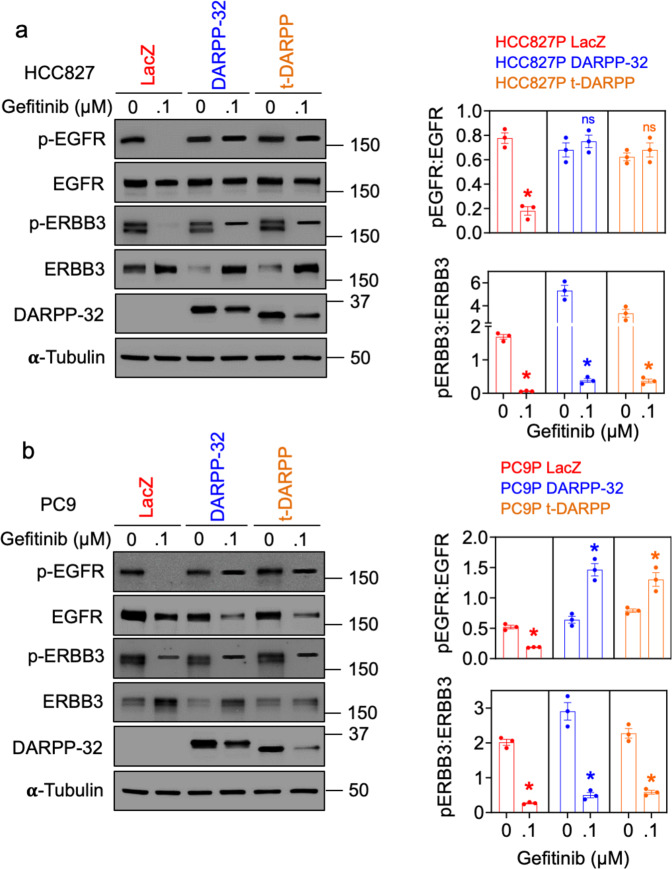
Overexpression of DARPP-32 isoforms increases phosphorylation of EGFR in the presence of gefitinib. **a**, **b** Human lung cancer cell line HCC827P (**a**) and PC9P (**b**) transduced with retrovirus containing control (LacZ), DARPP-32- or t-DARPP-overexpressing clones were treated with either vehicle (i.e., DMSO, 0 µM) or 0.1 µM gefitinib for 24 h. Cell lysates were separated in SDS-PAGE followed by immunoblotting analysis using primary antibodies against p-EGFR, EGFR, p-ERBB3, ERBB3, DARPP-32, and α-tubulin. Densitometry of western blots using ImageJ software was performed and band intensities were plotted as an average intensity of three independent western blot experiments. *P ≤ 0.05, 2-way unpaired *t*-test.

Given that DARPP-32 is overexpressed and ERBB3 is activated during gefitinib treatment in EGFR-mutated NSCLC cells, we propose a mechanism of acquired resistance to EGFR TKIs in NSCLC in which DARPP-32 mediates a switch from EGFR TKI-sensitive EGFR homodimers to TKI-resistant EGFR:ERBB3 heterodimers. This hypothesis is supported by findings showing that the physical association of EGFR and ERBB3 promotes resistance to gefitinib in NSCLC [55]. To test this hypothesis, we assessed the phosphorylation status of EGFR and ERBB3 proteins by immunoblotting in EGFR TKI-sensitive human NSCLC parental cells, HCC827 and PC9, overexpressing DARPP-32 or t-DARPP upon treatment with EGFR TKIs. Gefitinib-treated parental cells (i.e., HCC827P and PC9P) expressing DARPP-32 isoforms show higher p-EGFR expression compared to LacZ expressing cells (Fig. 3a, b). This trend of DARPP-32 isoform overexpression positively correlating with increased p-EGFR expression is maintained in HCC827P cells treated with osimertinib, a third-generation EGFR TKI (Supplementary Fig. 9a). However, DARPP-32 isoform overexpression does not affect p-EGFR levels in PC9P cells exposed to osimertinib (Supplementary Fig. 9b). We speculate that the irreversible EGFR-binding nature of osimertinib may contribute to DARPP-32 overexpression failing to mediate p-EGFR increases in osimertinib-treated PC9P cells, while the reversible binding of gefitinib to EGFR protein may enable DARPP-32-mediated elevations in p-EGFR in gefitinib-treated PC9P cells. Upon exposure to either gefitinib or osimertinib, we observed an increase of p-ERBB3 expression in HCC827P cells overexpressing DARPP-32 isoforms; however, no DARPP-32-mediated changes in p-ERBB3 expression were observed in the presence of either EGFR TKI in PC9 cells (Fig. 3a, b and Supplementary Fig. 9a, b). Based on our immunofluorescence studies, we observed a substantial reduction of p-EGFR intensity in gefitinib-treated LacZ-overexpressed control PC9P cells, whereas p-EGFR intensity in EGFR-mutated cells overexpressing DARPP-32 isoforms remained unchanged upon gefitinib treatment (Supplementary Fig. 10a, b). Overexpression of DARPP-32 and t-DARPP promotes increased p-ERBB3 upon EGFR TKI treatment (Supplementary Fig. 10a, c), suggesting that DARPP-32 upregulation may be associated with increased activation of ERBB3 in the presence of EGFR TKIs. We next asked whether stable shRNA-mediated depletion of DARPP-32 in gefitinib-resistant PC9GR3 cells affects phosphorylation of ERBB3 and EGFR upon gefitinib treatment. We observed a decrease in p-ERBB3 expression in gefitinib-treated DARPP-32-ablated PC9GR3 cells, whereas changes in p-ERBB3 levels were not detectable in corresponding LacZ shRNA control PC9GR3 cells upon treatment with gefitinib (Supplementary Fig. 11a, c). Others have reported that p-EGFR is not responsive to gefitinib in PC9GR3 cells [53]. Correspondingly, knockdown of DARPP-32 in gefitinib-treated PC9GR3 cells did not affect p-EGFR levels (Supplementary Fig. 11a, b). Collectively, our results demonstrating changes in activation of ERBB3 upon DARPP-32 modulation in the presence of EGFR TKI support a model in which DARPP-32 contributes to ERBB3-driven “bypass signaling” to promote EGFR-mutated NSCLC cell survival.

### Physical association between EGFR and ERBB3 is positively regulated by DARPP-32 isoforms

**Fig. 4:**
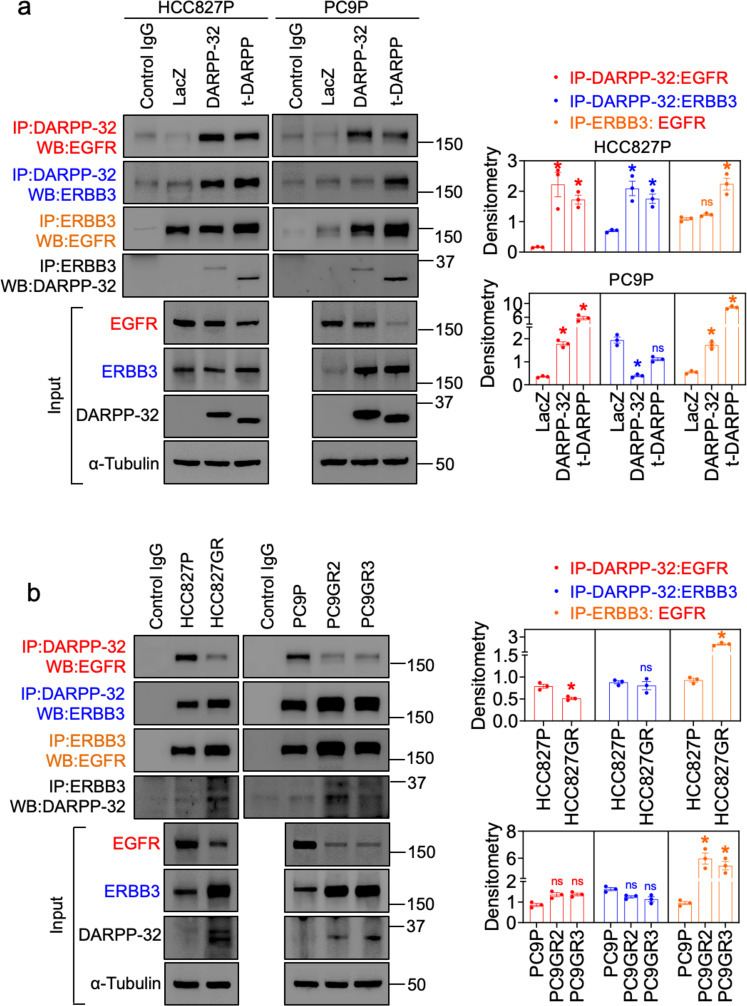
DARPP-32 physically associates with EGFR and ERBB3. **a** EGFR-mutated human NSCLC HCC827P and PC9P cells transduced with retrovirus encoding control (LacZ), DARPP-32- or t-DARPP-overexpressing clones were immunoprecipitated using antibodies against FLAG (detects both DARPP-32 isoforms) and ERBB3. Immunoprecipitated protein complexes and total cell lysates (input) were immunoblotted using EGFR, ERBB3, FLAG, and α-tubulin antibodies. **b** Human NSCLC HCC827P, HCC827GR, PC9P, PC9GR2, and PC9GR3 cells were lysed and immunoprecipitated with anti-DARPP-32 (recognizes endogenous DARPP-32 and t-DARPP) and anti-ERBB3 antibodies. Immunoprecipitated lysates along with total cell lysates were separated on SDS-PAGE followed by immunoblot analysis using antibodies against EGFR, ERBB3, DARPP-32, and α-tubulin. Immunoprecipitation experiments replicated three times were quantified using ImageJ software. Bar graphs represent mean ± SEM. *P ≤ 0.05, 2-way unpaired *t*-test ((**b**) top bar graph), 1-way ANOVA followed by Dunnett’s multiple comparison testing ((**a**) both bar graphs; (**b**) bottom bar graph).

To better understand the mechanism of resistance to gefitinib in EGFR-mutated NSCLC cells, we aimed to determine how DARPP-32 activates ERBB3 signaling to suppress gefitinib-mediated EGFR inhibition. To address our hypothesis that DARPP-32 drives EGFR:ERBB3 heterodimerization to evade EGFR TKI-mediated cell death, we performed immunoprecipitation studies to assess potential EGFR and ERBB3 interactions in DARPP-32-modulated EGFR-mutated NSCLC cells. Immunoprecipitation using an anti-ERBB3 antibody demonstrates that EGFR and ERBB3 physically interact and that the EGFR:ERBB3 association increases upon overexpression of t-DARPP in HCC827P cells and upon overexpression of both DARPP-32 isoforms in PC9P cells (Fig. 4a). Furthermore, immunoprecipitation for DARPP-32 in parental cells reveals that DARPP-32 physically interacts with EGFR and ERBB3 (Fig. 4a), suggesting it associates with the EGFR:ERBB3 complex. We sought to investigate how DARPP-32 affects EGFR and ERBB3 interactions in gefitinib-resistant cells relative to gefitinib-sensitive parental cells. ERBB3 immunoprecipitation experiments suggest that DARPP-32 upregulation results in increased association of EGFR with ERBB3 in gefitinib-resistant HCC827GR, PC9GR2, and PC9GR3 cells relative to their gefitinib-sensitive counterparts (Fig. 4b), suggesting that DARPP-32 may promote EGFR:ERBB3 heterodimer formation in EGFR TKI-resistant cells.

### DARPP-32-driven p-EGFR/p-ERBB3 heterodimerization activates downstream MEK/ERK and PI3K/AKT/mTOR signaling

**Fig. 5:**
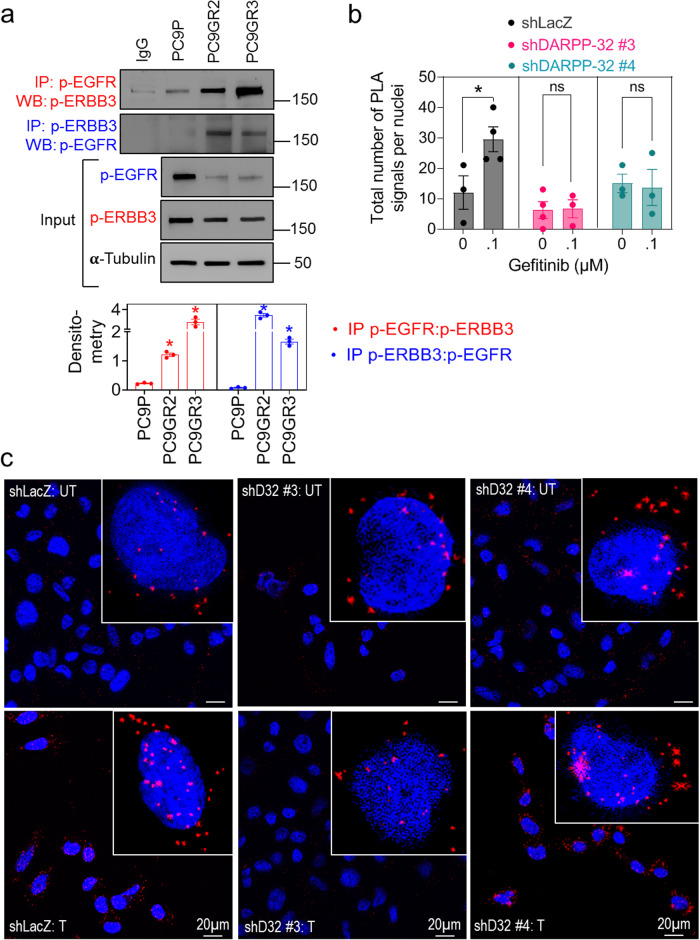
Depletion of DARPP-32 reduces p-EGFR to p-ERBB3 heterodimer formation. **a** Lysates of PC9P, PC9GR2, and PC9GR3 cells were used to perform immunoprecipitation experiments using p-EGFR and p-ERBB3 antibodies. Total cell lysates (input) as well as immunoprecipitated protein complexes were subjected to western blotting using primary antibodies against p-EGFR, p-ERBB3, and α-tubulin. Immunoblots representative of three independent experiments were quantified using ImageJ software. Data represent mean ± SEM. *P ≤ 0.05, 1-way ANOVA followed by Dunnett’s multiple comparison testing. **b** Proximity ligation assays (PLA) were performed using PC9GR3 cells stably transduced with control (shLacZ) or DARPP-32 shRNAs (shDP32) using antibodies against p-ERBB3 and p-EGFR following 24 h incubation with either vehicle (UT) or 100 nM gefitinib (T). Total number of PLA signals per cell have been reported after calculating red fluorescence signals of 6–10 random microscopic fields for each group. Each circle on a graph represents an independent experiment. Bar graphs represent mean ± SEM of three independent experiments. *P < 0.05, 2-way unpaired *t*-test**. c** The images show a maximum intensity projection of the raw image based on 10 *z*-planes. PLA signals are shown in red and the DAPI-stained nuclei in blue. Magnified images of a single cell are depicted in the inset. Scale bar, 20 µm.

**Fig. 6:**
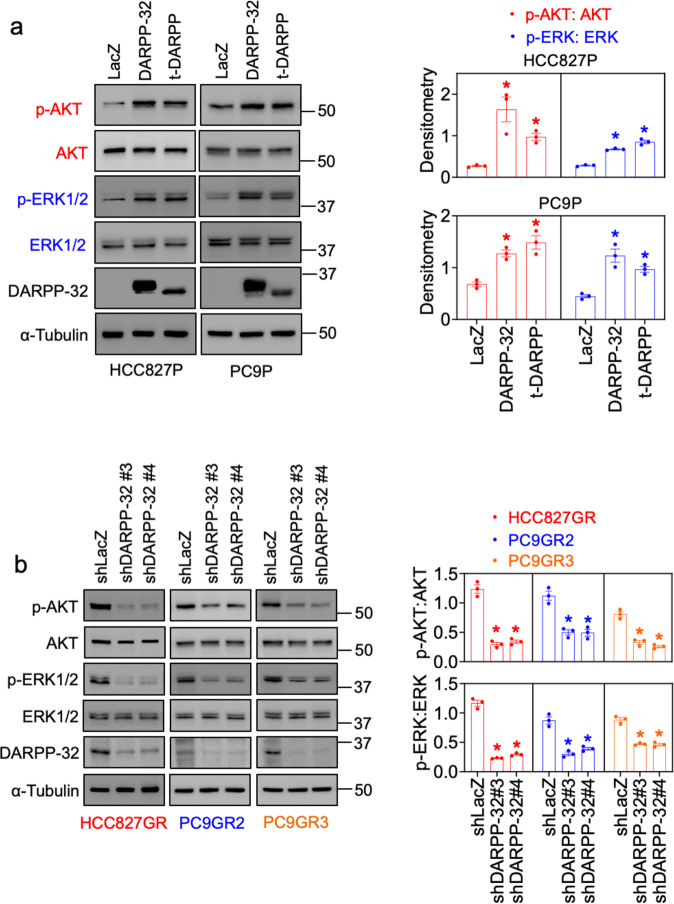
DARPP-32 activates AKT and ERK signaling in the presence of gefitinib. **a** Immunoblotting was performed in gefitinib-treated HCC827P and PC9P cells stably overexpressing control (LacZ), DARPP-32, or t-DARPP using antibodies against phosphorylated AKT (p-AKT), total AKT (AKT), phosphorylated ERK (p-ERK1/2), total ERK (ERK1/2), DARPP-32, and α-tubulin (loading control). **b** Gefitinib-resistant human lung cancer cell lines, HCC827GR, PC9GR2, and PC9GR3, were transduced with lentivirus encoding LacZ or DARPP-32 shRNAs and treated with 100 nM gefitinib for 24 h. Cell lysates were separated and antibody-reactive protein bands were detected using p-AKT, AKT, p-ERK, ERK, DARPP-32, and α-tubulin antibodies. Three independent immunoblotting experiments have been performed and representative results from one experiment have been shown. Densitometric analysis of the immunoblots depicted in bar graphs has been performed to quantify the expression of p-AKT and p-ERK relative to their respective total protein expression. Each circle represents an independent experiment. Error bars indicate SEM. *P ≤ 0.05, 1-way ANOVA followed by Dunnett’s multiple comparison testing.

Given that ERBB3 has limited kinase activity and relies on heterodimerization with EGFR for activation [56], we postulate that DARPP-32 promotes ERBB3 phosphorylation by increasing physical association between p-EGFR and p-ERBB3. To address our theory, we first performed immunoprecipitation experiments detecting physical association between p-EGFR and p-ERBB3 proteins in parental and gefitinib-resistant PC9 cells. We observed the physical association between p-EGFR and p-ERBB3 proteins markedly increases in PC9GR2 and PC9GR3 cells relative to their parental equivalent (Fig. 5a). We next performed a proximity ligation assay (PLA) using anti-p-EGFR and anti-p-ERBB3 antibodies in PC9GR3 cells. PLA is a powerful tool for identifying protein–protein interaction in situ with high specificity and sensitivity. Our PLA findings suggest that gefitinib treatment induces p-EGFR/p-ERBB3 heterodimer complex formation in PC9GR3 cells (Fig. 5b, c). However, ablation of DARPP-32 in PC9GR3 cells abolishes gefitinib-induced p-EGFR/p-ERBB3 dimerization, suggesting that DARPP-32 plays a significant role in the formation of these active heterodimers (Fig. 5b, c). We next sought to determine how DARPP-32 regulates MEK/ERK and PI3K/AKT signaling pathways in the presence of gefitinib. It has been reported that ligand-independent EGFR activation initiates intracellular signaling via Ras/Raf/MEK/ERK and PI3K/AKT signaling pathways [13]. By immunoblotting we show that overexpression of DARPP-32 isoforms increases p-AKT and p-ERK expression in gefitinib-treated sensitive cells (Fig. 6a). Knockdown of DARPP-32 reduces p-AKT and p-ERK expression in gefitinib-treated resistant cells (Fig. 6b). EGFR-dependent PI3K activation requires dimerization with the ERBB3 receptor because docking sites of PI3K (i.e., p85 subunit) are abundant on ERBB3 and absent within EGFR [13]. To test our hypothesis that DARPP-32 activates the PI3K signaling pathway in EGFR-mutated NSCLC cells, we used a bioinformatics approach to assess DARPP-32 transcript expression in specimens derived from 80 EGFR-mutated LUAD patients cataloged in The Cancer Genome Atlas (TCGA). We first subdivided LUAD patient-derived specimens into two groups based on high versus low DARPP-32 mRNA expression (Supplementary Fig. 12a). Interestingly, we found that expression of RPS6KB2 transcripts, but not RPS6KB1 transcripts, increases in lung tumor specimens with high expression of DARPP-32 (Supplementary Fig. 12b, c). The ribosomal S6 kinase isoforms (i.e., RPS6KB1, RPS6KB2) are downstream targets of PI3K/AKT/mTOR signaling [57]. Given that both kinases phosphorylate the 40S ribosomal protein S6 [57], we next assessed the expression of phosphorylated RPS6 proteins to determine whether increased expression of RPS6KB2 affects the phosphorylation status of RPS6. Our results reveal increased expression of phospho-RPS6 proteins (i.e., pS235/S236 and pS240/S244) in the LUAD patient-derived specimens with high DARPP-32 transcript levels. The expression of unmodified (i.e., total) RPS6 protein among high versus low DARPP-32 transcript groups is unchanged, suggesting that upregulation of RPS6KB2 results in RPS6 protein activation (Supplementary Fig. 12d–f). Taken together, our findings suggest DARPP-32 promotes dimerization of active EGFR and ERBB3 receptors to stimulate PI3K/AKT/mTOR and MEK/ERK signaling in EGFR TKI-refractory NSCLC progression.

### DARPP-32 promotes EGFR TKI-refractory tumor growth in vivo

**Fig. 7:**
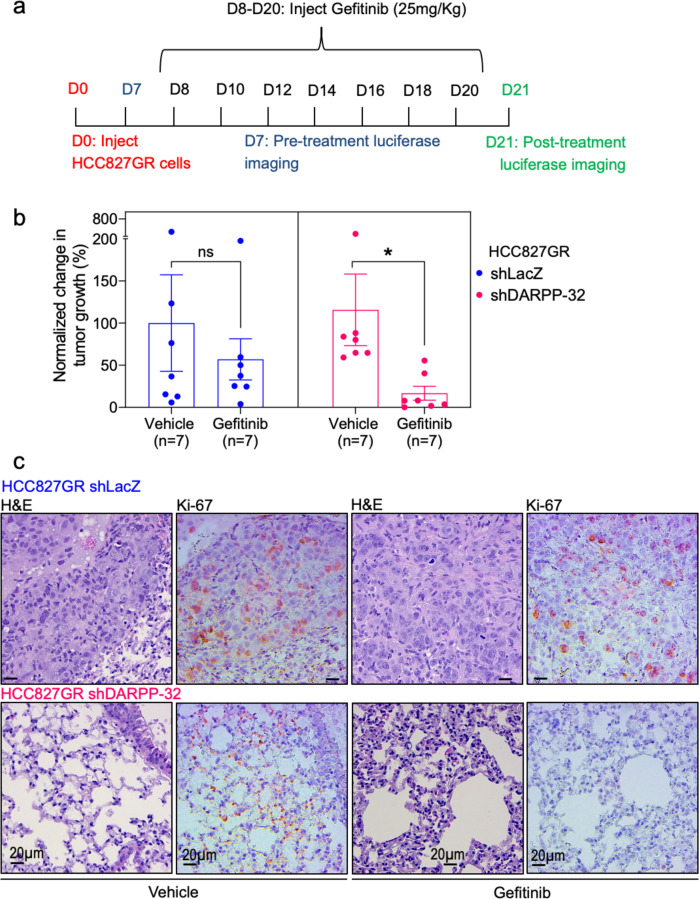
DARPP-32 silencing reduces EGFR TKI-refractory tumor growth in vivo. **a** Luciferase-labeled human HCC827GR cells transduced with control (LacZ) or DARPP-32 shRNA were injected into the left thorax of SCID mice (*n* = 7 mice per group), imaged for luminescence, administered 25 mg/kg gefitinib on indicated days, and re-imaged at the indicated experimental endpoint. **b** Quantification of tumor growth was reported by determining the difference in relative luciferase units (RLU) before and after drug treatment. Bar graphs represent mean ± SEM. *P < 0.05, 2-way unpaired *t*-test**. c** Immunohistochemistry was performed using monoclonal Ki-67 antibody on formalin‐fixed, paraffin‐embedded lung tissue (*n* = 3 mice per group) obtained from human lung tumor xenograft model mice. For evaluation of the histopathology, slides were stained with hematoxylin and eosin (H&E) dye. Scale bar, 20 µm.

Based on our findings suggesting that DARPP-32 increases ERBB3 phosphorylation to bypass gefitinib-induced EGFR inhibition, we next sought to understand whether DARPP-32 drives NSCLC resistance to EGFR TKIs in vivo. To this end, we tested whether DARPP-32 ablation increases EGFR TKI sensitivity in a gefitinib-resistant orthotopic xenograft mouse model. Briefly, we injected luciferase-labeled human gefitinib-resistant (HCC827GR) NSCLC cells into the left thorax of anesthetized SCID mice, confirmed establishment of lung tumors via non-invasive luciferase imaging, and administered gefitinib over the course of 2 weeks, and measured tumors through luciferase imaging (Fig. 7a). Mice challenged with HCC827GR cells with DARPP-32 stably silenced by shRNA show decreased tumor growth when treated every other day with gefitinib relative to vehicle controls (Fig. 7b and Supplementary Fig. 13a). DARPP-32 knockdown sensitizes gefitinib-resistant NSCLC tumors to EGFR inhibition in vivo, whereas no such effect was observed in mice challenged with control LacZ shRNA-transduced HCC827GR cells (Fig. 7b and Supplementary Fig. 13a). Histological sections from these mice were immunostained for Ki-67. We observed decreased tumor cell proliferation in the lungs of gefitinib-treated mice challenged with DARPP-32-silenced HCC287GR cells (Fig. 7c), confirming that DARPP-32 knockdown enhances EGFR TKI-induced anti-cancer effects in gefitinib-resistant tumors in vivo.

**Fig. 8:**
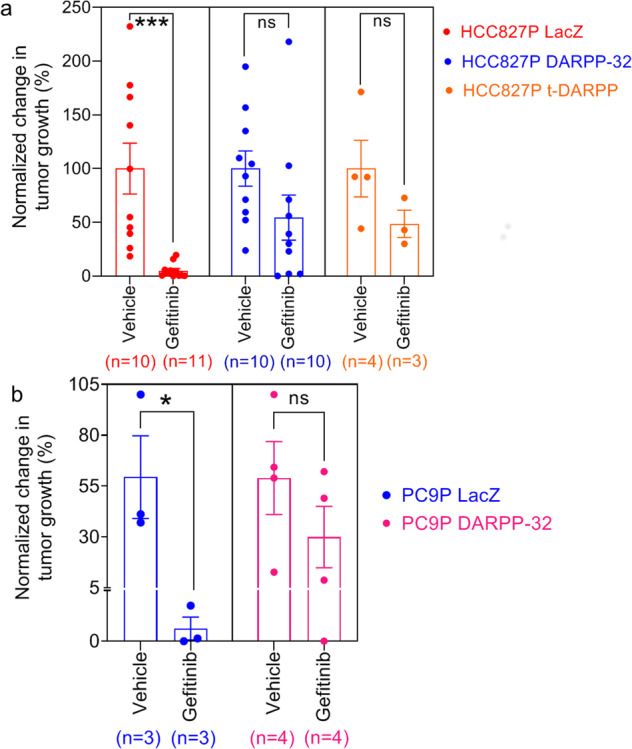
DARPP-32 overexpression protects EGFR TKI-sensitive human lung tumor xenografts from gefitinib-induced tumor reduction. **a** SCID mice were orthotopically injected with luciferase-labeled human HCC827P cells stably overexpressing control (LacZ), DARPP-32, or t-DARPP cDNAs. Vehicle- and gefitinib-treated mice were imaged for luminescence, and quantification of HCC827P tumor growth before and after treatment has been reported. Percentage change in tumor growth among two independent experiments has been reported. **b** Human lung cancer PC9P cells transduced with retrovirus containing cDNA plasmids designed to overexpress control (LacZ) or DARPP-32 proteins were injected subcutaneously into the right flank of SCID mice. Mice were administered either vehicle or gefitinib (25 mg/kg) via oral gavage three times in a week beginning once mean tumor volume had reached 150 mm^3^. Tumor growth was assessed by measuring tumor volume with calipers before and after drug treatment. Experiments were concluded before tumor volume exceeded 1500 mm^3^. Bar graphs represent mean ± SEM. Number of mice (*n*) used in each group has been reported at the bottom of bar graphs. *P < 0.05, ***P < 0.001, 2-way unpaired *t*-test.

We next sought to determine whether overexpression of DARPP-32 isoforms promotes resistance to gefitinib in vivo. Gefitinib-sensitive HCC827P tumors overexpressing DARPP-32 or t-DARPP that were implanted orthotopically into the lungs of mice exhibit gefitinib resistance relative to controls (Fig. 8a and Supplementary Fig. 13b). To confirm the role of DARPP-32 in EGFR TKI resistance using a different NSCLC xenograft model, gefitinib-sensitive PC9P cells stably overexpressing DARPP-32 were subcutaneously injected into the flank of SCID mice. Once tumors reached ~150 mm^3^, mice were treated with EGFR TKI, gefitinib. Mice harboring DARPP-32 overexpressing PC9P tumors do not respond to gefitinib (Fig. 8b and Supplementary Fig. 14a), suggesting that DARPP-32 promotes resistance to gefitinib. Extirpated tumor volume and weight measurements confirm this finding (Supplementary Fig. 14b, c). Collectively, our observations demonstrate that DARPP-32 promotes gefitinib-refractory tumor growth in vivo. Taken together with our in vitro findings, our results indicate that DARPP-32 isoforms promote EGFR TKI-refractory NSCLC cell survival by stimulating the formation of active EGFR and ERBB3 heterodimers, increased AKT and ERK activation, and evasion of EGFR TKI-dependent apoptosis.

### Elevated DARPP-32 expression is associated with EGFR TKI resistance in NSCLC patients

**Fig. 9:**
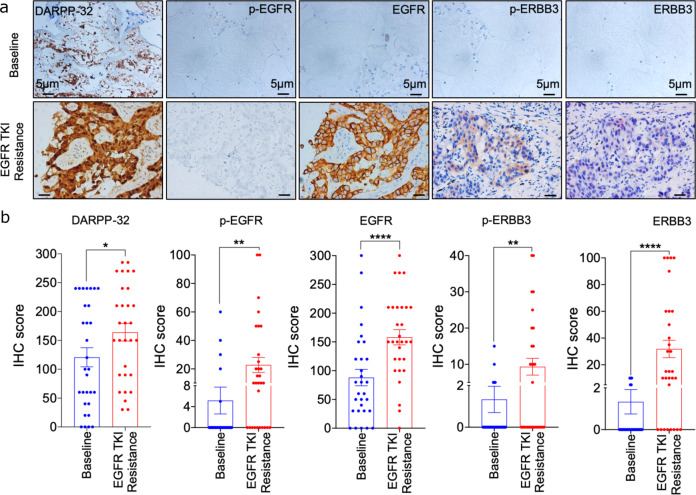
LUAD patients exhibit increased DARPP-32, p-EGFR, and p-ERBB3 expression upon EGFR TKI-refractory disease progression. **a** Paired lung tumor biopsies were obtained from LUAD patients with EGFR activating mutations (*n* = 30 patients) before EGFR TKI treatment (i.e., baseline) and following EGFR TKI resistance (i.e., progressive disease after first-line gefitinib or erlotinib therapy). Paired baseline (top) and EGFR TKI resistance (bottom) lung tumor specimens were immunostained for DARPP-32, phosphorylated EGFR (p-EGFR), total EGFR, p-ERBB3, and total ERBB3. **b** IHC score was calculated by multiplying the staining intensity score (0–3) by the percent of positive tumor cells. Each circle on the plots represents single patient. *P < 0.05, **P < 0.01, ^****^P < 0.0001, 2-way unpaired *t*-test.

To investigate the clinical relevance of DARPP-32 given its role in promoting resistance to EGFR first-generation TKIs in mouse models of human NSCLC, we assessed DARPP-32, p-EGFR, total EGFR, p-ERBB3, and total ERBB3 protein expression by immunostaining in paired EGFR TKI-naive and -resistant specimens from 30 LUAD patients (Supplementary Table 1). Briefly, lung tumor specimens were biopsied from LUAD patients before EGFR TKI treatment (i.e., baseline) and following the development of progressive disease after first-line gefitinib or erlotinib therapy. For immunostaining of each protein, three pathologists independently scored the percentage of tumor cells staining positive and corresponding staining intensity (i.e., 0 = none, 1 = weak, 2 = moderate, 3 = strong expression). We calculated an immune-reactive (IR) score for each specimen based on the percentage of tumor cells staining positive and the staining intensity in those cells (IR score = percentage of tumor cells × staining intensity). We found that DARPP-32, kinase-activated EGFR, total EGFR, kinase-activated ERBB3, and total ERBB3 proteins are upregulated in first-generation EGFR TKI-resistant NSCLC patient-derived specimens relative to individual patient-matched (i.e., paired) baseline samples biopsied prior to frontline gefitinib or erlotinib treatment (Fig. 9a, b). Based on molecular profiling of the 30 specimens acquired from patients during EGFR TKI-refractory progression, we identified that most specimens harbored EGFR^T790M^ mutations (*n* = 17), whereas the other specimens had either unknown alterations (*n* = 9), MET amplifications (*n* = 2), KRAS G12D mutation (*n* = 1), or MYC amplification and TP53 mutation (*n* = 1; Supplementary Table 1). We assessed whether elevated DARPP-32 protein expression correlated with specific resistance mechanisms, such as EGFR^T790M^ mutations. However, we observed no statistically significant difference in DARPP-32 protein expression in specimens from patients carrying EGFR^T790M^ mutations (*n* = 17) relative to cumulative group of other alterations (*n* = 13; Supplementary Fig. 15). Collectively, our results suggest that DARPP-32 overexpression and increased EGFR and ERBB3 activation is associated with EGFR TKI resistance in NSCLC patients.

## Discussion

Lung cancer is the deadliest and most frequently diagnosed type of tumor worldwide, with 1.6 million deaths reported annually [58]. The molecular targeting of specific oncogenic drivers has emerged as a major advancement in the treatment of NSCLC. Patients diagnosed with advanced non-squamous cell NSCLC are tested for oncogenic alternations and treated accordingly [20, 59]. Single oncogenic driver mutations in EGFR that confer sensitivity to TKIs are the most common targetable molecular alteration in LUAD. Although EGFR mutation-positive patients initially respond well to EGFR TKI therapy, most patients inevitably develop resistance and experience rapid advanced-disease progression. Developing acquired resistance to lung cancer therapy is a major problem. The development of effective strategies to circumvent the emergence of this resistance is needed to improve survival rates and the quality of life of NSCLC patients.

The spectrum of identified EGFR resistance mechanisms includes on-target EGFR gatekeeper mutations (i.e., EGFR^T790M^), amplifications of MET and ERBB2, MAPK-PI3K signaling activation, cell cycle alterations, rearrangements of RET or ALK kinases, and various other genomic alternations [60–62]. Here, we sought to better understand the molecular mechanisms that control aberrant ERBB family signaling that drives EGFR TKI-refractory LUAD progression. ERBB receptor tyrosine kinase family members, including ERBB1-ERBB4 (also known as EGFR, HER2, HER3, and HER4), consist of a single hydrophobic transmembrane region flanked by an extracellular ligand-binding domain and an intercellular tyrosine kinase domain [14]. ERBB family member signaling activates interconnected pathways that promote oncogenesis, including PI3K/AKT/mTOR and MAPK signal transduction [63, 64], as well as PLCγ/PKC [65, 66] and JAK2/STAT3 pathways [67, 68]. ERBB3, specifically, has been implicated in the initiation of EGFR TKI resistance. Unlike its fellow family members, ERBB3 was initially believed to be an inactive kinase because its kinase domain lacks certain residues known to be essential for catalytic activity [69]. However, ERBB3 forms heterodimers with other ERBB family members to become transphosphorylated and transactivated to sustain transduction of downstream oncogenic signaling that would otherwise be inhibited by EGFR TKIs acting upon EGFR homodimers [70–72]. Several known mechanisms of ERBB3-induced TKI resistance exist, by which ERBB3 compensates for TKI-inhibited EGFR to trigger and sustain PI3K/AKT signal transduction. First, MET amplification has been shown to result in constitutive activation of ERBB3 signaling to promote gefitinib resistance in lung cancer cell lines [52]. Second, ERBB3 heterodimerization with ERBB2 has been demonstrated to drive oncogenic signaling in breast cancer [73] as the effects of ERBB2 inhibition could be reversed by increasing ERBB3 phosphorylation and activity to drive a TKI resistance phenotype [72]. Third, ligand-mediated activation of ERBB3 has been shown to result in PI3K/AKT-mediated resistance to TKIs in a variety of cancers, including ERBB2-amplified breast cancer cells stimulated with ERBB3 ligands, NRG1 [74] or HRG [75]. We identify a new mechanism of ERBB3-mediated TKI resistance in which DARPP-32 physically stimulates this process of EGFR:ERBB3 heterodimer formation to promote PI3K/AKT and MAPK signaling to overcome the inhibitory effects of EGFR TKIs.

We were the first to report that DARPP-32 overexpression in lung cancer contributes to oncogenic growth [31]. While DARPP-32 is virtually undetectable in normal human lung [29], DARPP-32 is overexpressed in human EGFR-mutated NSCLC. Specifically, we previously demonstrated that DARPP-32 proteins promote NSCLC cell survival through increased AKT and ERK1/2 signaling [31]. Given that these PI3K and MAPK signaling pathways are upregulated during resistance, we hypothesized that overexpression of DARPP-32 proteins in EGFR-mutated NSCLC may promote EGFR:ERBB3 “bypass signaling” that enables tumor cells to evade EGFR TKI monotherapy. In this report, we provide the first evidence that DARPP-32 overexpression in EGFR-mutated LUAD promotes ERBB3-mediated oncogenic signaling to drive EGFR TKI therapy-refractory cancer progression. In vivo studies reveal that ablation of DARPP-32 protein activity sensitizes gefitinib-resistant lung tumor xenografts to EGFR TKI treatment, while DARPP-32 overexpression increases gefitinib-refractory lung cancer progression in gefitinib-sensitive lung tumors orthotopically xenografted into mice. Findings from proximity ligation assays, immunoprecipitation studies, and immunofluorescence experiments presented here support a model in which DARPP-32 mediates a switch from EGFR TKI-sensitive EGFR homodimers to TKI-resistant EGFR:ERBB3 heterodimers to potentiate oncogenic AKT and ERK signaling that drives therapy-refractory tumor cell survival. To our knowledge, no proteins have been identified that are capable of mediating such a “dimerization switch” in EGFR-mutated NSCLC. Here, we take advantage of a unique cohort of paired tumor specimens derived from 30 LUAD patients before and after the development of EGFR TKI-refractory disease progression to reveal that DARPP-32 as well as kinase-activated EGFR and ERBB3 proteins are overexpressed upon acquired EGFR TKI resistance. This observation coincides with our published report that increased t-DARPP immunostaining positively correlates with increasing T stage among unknown EGFR mutation status NSCLC patients [31]. There is no precedent of DARPP-32 isoform immunostaining in molecular targeted therapy naive vs. resistant patients in other tumor types.

While it is well-established that ERBB3 plays a role in EGFR TKI resistance [70–72], ERBB3-independent activation of the PI3K/AKT pathway has been demonstrated in EGFR-mutated LUAD [76]. Using transgenic mouse models, Dr. Politi’s group showed that ERBB3 is not required for mutant EGFR-induced tumorigenesis [76]. However, acute loss of ERBB3 inhibited growth of established EGFR^L858R^ mutation-driven tumors in mice. Tumors that developed in the absence of ERBB3 continued to exhibit activation of PI3K/AKT signaling and retained sensitivity to the EGFR TKI erlotinib [76]. Activation of PI3K/AKT pathway by EGFR occurs through two distinct mechanisms: (1) EGFR heterodimerizing with ERBB3 [77] or (2) EGFR directly recruiting adapter molecules, such as GAB1 and GAB2, that couple with p85 [78]. Here, we demonstrate that DARPP-32 promotes the formation of EGFR:ERBB3 heterodimers to potentiate oncogenic PI3K/AKT signaling. While beyond the scope of this current study, it is possible that DARPP-32 also contributes to ERBB3-independent PI3K/AKT activation in EGFR-mutated LUAD. In small cell lung cancer, NOTCH inactivation leads to ASCL1-mediated upregulation of DARPP-32 and activation of AKT/ERK signaling [29] that is likely independent of the EGFR-ERBB3 axis, suggesting that DARPP-32 may promote tumor cell survival through multiple mechanisms. In breast cancer cells, t-DARPP protein associates with IGF-1R resulting in increased AKT phosphorylation and greater glycolysis [47]. Additionally, t-DARPP promotes cancer cell survival in the presence of trastuzumab by stimulating elevated levels of the anti-apoptosis protein Bcl-2, which leads to increased phosphorylation of AKT [44]. Here, we report that DARPP-32 promotes gefitinib-refractory survival of human EGFR-mutated LUAD cells (Fig. 2). Future studies are necessary to determine how DARPP-32 differentially regulates ERBB3-independent and -dependent lung tumor cell survival.

Hyperactive PI3K/AKT signaling has been shown to be a major mechanism of resistance to EGFR TKIs, and the AKT/mTOR inhibitor dactolisib has recently been shown to circumvent resistance when administered in combination with osimertinib both in vitro and in vivo [79]. While the precise mechanism by which DARPP-32 activates AKT signaling is unknown, it has been widely shown that DARPP-32 mediates increases in AKT signaling [29, 31, 38, 45–47, 49, 80–84]. Given that PI3K/AKT signaling is likely activated as a result of both ERBB3-independent and -dependent resistance mechanisms, targeting the PI3K/AKT pathway in cases of DARPP-32-mediated resistance represents a plausible strategy. Direct targeting of DARPP-32 has been hindered by its lack of known tertiary structure. The acidic glutamine- and aspartate-rich regions of DARPP-32 might prohibit crystallization of their elongated monomer secondary structure, consisting of 12% alpha helices, 29% beta strands, 24% beta turns, and 35% random coils [85]. However, promising new methods have recently emerged using antisense oligonucleotides to inhibit the function of previously “undruggable” targets [86], including YAP1 [87]. Use of antisense oligonucleotides to target DARPP-32 warrants preclinical investigation. The changes in EC50s in the presence of gefitinib upon DARPP-32 modulation are small relative to EC50 differences that would likely be observed in EGFR-mutated cells in which MET is overexpressed or a kinase fusion is introduced to drive resistance (Supplementary Fig. 1). However, these relatively small DARPP-32-mediated changes are important because resistance clones can build up slowly over time.

Here, we used two models of human EGFR-mutated LUAD resistance with distinct gefitinib resistance mechanisms. PC9GR-2 and -3 cells harbor an EGFR^T790M^ mutation, whereas bypass signaling through MET confers EGFR TKI resistance in HCC827GR cells. Our findings suggest that DARPP-32 promotes ERBB3-mediated resistance to EGFR TKIs in both models despite their differing molecular mechanisms of resistance. Therefore, we speculate that established resistance mechanisms, such as EGFR^T790M^ and MET amplifications, may promote overexpression of DARPP-32 proteins, leading to enhanced EGFR:ERBB3 heterodimer formation. This hypothesis is supported by our finding that DARPP-32 protein is upregulated upon EGFR TKI-refractory progression in a cohort of paired patient specimens before and after the development of resistance (Fig. 9), but this theory certainly requires additional future investigation. It is also conceivable that DARPP-32 might contribute to TKI therapy-refractory growth in adenocarcinomas in which additional downstream genetic alterations exist in genes like RET, ALK, BRAF, or KRAS, although such studies are yet to be conducted.

Our data collectively suggest that DARPP-32 overexpression promotes EGFR TKI resistance by stimulating formation of EGFR:ERBB3 heterodimers, which are less sensitive to EGFR inhibition and drive oncogenic signaling. Therefore, dual inhibition of EGFR and ERBB3 may better prevent treatment-refractory cancer progression as opposed to solely targeting EGFR, especially in tumors overexpressing DARPP-32. A precision oncology approach could be used to identify EGFR-mutated LUADs with high DARPP-32 and phosphorylated ERBB3 expression with the highest likelihood to benefit from dual EGFR and ERBB3 inhibition. For example, duligotuzumab is a human IgG1 monoclonal “two-in-one” antibody with high affinity for EGFR (*K*_D_ ~ 1.9 nM) and ERBB3 (*K*_D_ ~ 0.4 nM) developed to improve treatment response of solid tumors exhibiting ERBB3-mediated resistance to EGFR-targeted treatment [88]. Partial responses to duligotuzumab were achieved in patients with squamous cell carcinoma of the head and neck that had become resistant to cetuximab, an antibody therapy that inhibits EGFR [89]. Efficacy was also observed in tumors’ refractory to both radiation and long-term EGFR-targeted treatment [90, 91]. Duligotuzumab monotherapy has been shown to be well-tolerated in patients with locally advanced or metastatic solid tumors of epithelial origin [89]. However, a recent randomized phase II study of duligotuzumab vs. cetuximab in squamous cell carcinoma of the head and neck (i.e., MEHGAN; NCT01577173) found duligotuzumab did not improve disease-free survival compared to cetuximab [92]. However, poor patient selection may have confounded its outcome as suggested by Dr. Saba in an associated Commentary [93]. Regardless, dual inhibition of EGFR and ERBB3 warrants further clinical investigation in trials that focus specifically on EGFR treatment-resistant patient populations [94]. Such clinical trials have not been performed and nor have any duligotuzumab studies in the clinic focused on EGFR-mutated LUAD patients. Future studies evaluating a dual EGFR and ERBB3 inhibitory approach in models of acquired EGFR TKI resistance are warranted, given that DARPP-32-mediated, ERBB3-driven resistance is a key mechanism of EGFR TKI-refractory LUAD progression, combined with the well-established safety profile of duligotuzumab.

## Methods

Please see Supplementary Methods for methodological descriptions of generation of stable cell lines, antibodies, immunoblotting, cell survival assay, apoptosis assay, immunofluorescence, immunoprecipitation, proximity ligation assay, gene expression analysis, and immunohistochemistry.

### Cell culture

Gefitinib-sensitive HCC827 parental (HCC827P) and HCC827 gefitinib-resistant (HCC827GR) human NSCLC cell lines were a generous gift from Dr. Pasi Jänne [52]. Gefitinib-sensitive PC9 parental (PC9P) cells and their gefitinib-resistant counterparts (PC9GR2, PC9GR3) were kindly provided by Dr. Aaron Hata [53]. HCC827P and PC9P cells were grown in RPMI-1640 medium (Corning). RPMI-1640 medium containing 1 µm of gefitinib (Selleckchem) was used to culture HCC827GR, PC9GR2, and PC9GR3 cells. HEK-293T cells were purchased from ATCC and maintained in DMEM (Corning). All media were supplemented with 10% fetal bovine serum (FBS; Millipore), 1% penicillin/streptomycin antibiotics (Corning), and 25 µg/ml Plasmocin prophylactic (Invivogen). All cell lines were authenticated by their source and were subsequently routinely authenticated via morphologic inspection.

### In vivo orthotopic lung cancer model

Six- to eight-week-old pathogen-free SCID/NCr mice purchased from the Charles River Laboratories were maintained in accordance with protocols approved by the University of Minnesota Institutional Animal Care and Use Committee (IACUC). Mice were allowed 1 week to acclimate to their surroundings, bred, maintained under specific pathogen-free (SPF) conditions in a temperature-controlled room with alternating 12 h light/dark cycles, and fed a standard diet. Eight- to twelve-week-old male and female mice were anesthetized with pharmaceutical-grade ketamine (90–120 mg/kg) and xylazine (5–10 mg/kg) via intraperitoneal injection using a laminar flow hood in an SPF room within the animal facility. Each fully anesthetized mouse was placed in the right lateral decubitus position and the left lateral chest was sterilized. One-million luciferase-labeled human HCC827GR and HCC827P lung cancer cells suspended in 80 μl PBS and high-concentration Matrigel (Corning; Cat. no.: 354248) were orthotopically injected in the left thoracic cavity of each mouse. Based on the captured luminescence images of mice using an In-Vivo Xtreme Xenogen Imaging System (Bruker) as described [29], mice were randomly divided into two groups with similar average luminescence intensities. After establishment of the lung tumor, mice were administered either vehicle or gefitinib (25 mg/kg) every other day for 2 weeks. Upon completion of the study, mice were euthanized using asphyxiation by CO_2_ inhalation to effect with a flow rate displacing less than 30% of the chamber volume per minute in accordance with IACUC euthanasia guidelines and consistent with recommendations of the Panel of Euthanasia of the American Veterinary Medical Association. Following euthanasia, lungs were perfused, harvested, and portions the lungs from each mouse were preserved in formalin for immunohistochemistry, RNAlater Stabilization Solution (Thermo Fisher Scientific) for RNA extraction, and flash frozen for protein extraction. Bruker molecular imaging software was used to calculate luciferase intensity (total photons count) of tumor cells in each mouse. Tumor growth was determined by plotting average luciferase intensity over time in GraphPad Prism 8 software.

### In vivo subcutaneous lung tumor model

Eight- to twelve-week-old male and female SCID/NCr mice were subcutaneously injected with 2 × 10^6^ luciferase-labeled human PC9P lung cancer cells suspended in 80 μl PBS and high-concentration Matrigel. To determine tumor growth, the tumor volume was measured every week using the formula: (length × width^2^)/2. After establishment of palpable tumor (≥150 mm^3^), mice were randomly divided into two groups and administered either vehicle or gefitinib (25 mg/kg). At the endpoint, mice were euthanized by CO_2_ asphyxiation. Extirpated tumors were photographed, weighed, and preserved in formalin for immunohistochemistry analysis. All animal studies were approved by IACUC, and investigators were not blinded to animal group allocation.

### Clinical workflow and patient selection

Patients who met the following criteria were enrolled in this study: (1) pathologically confirmed advanced LUAD; (2) NGS identified EGFR exon 21 L858R mutation; (3) treatment with gefitinib or erlotinib in the first-line setting; and (4) accessed with disease progression and available tumor sample at baseline and progression. Patients were examined every 2 weeks after EGFR TKI administration and 20% incensement of tumor burden is considered as disease progression according to RECIST 1.1. Pathological diagnosis and staging were carried out according to the staging system of the 2009 International Association for the Study of Lung Cancer (version 8). Written informed consent was obtained from all the patients prior to inclusion to this study. Approval was also obtained from Hunan Cancer Hospital Institutional Review Board (IRB) Committee.

### Statistics

To compare differences between two groups, two-way unpaired *t*-tests were performed and values of *P* ≤ 0.05 were considered significant. One-way analysis of variance (ANOVA) followed by Dunnett’s test was used to determine statistically significant differences between multiple groups (greater than two). Data expressed as mean ± SEM are representative of at least three independent experiments. For most animal experiments, the number of animals per group was calculated based on a one-way ANOVA analysis to allow 90% power when the mean in the test group is 1.25 standard deviations higher or lower than the mean in the controls.

## Supplementary information

Supplementary Figures 1–15 and Supplementary Table 1.

Supplementary methods.

## Data Availability

The authors declare that the data supporting the findings of this study are available within the article and its supplementary information.
