# Supplementary material for: DARPP-32 promotes ERBB3-mediated resistance to molecular targeted therapy in EGFR-mutated lung adenocarcinoma
Source: Oncogene. 2021 Oct 21;41(1):83–98. doi: 10.1038/s41388-021-02028-5 (PMC8529229; doi:10.1038/s41388-021-02028-5)
Supplement: Supplementary file 1 — Supplementary Figures 1–15 and Supplementary Table 1. [file 41388_2021_2028_MOESM1_ESM.pdf]

Supplementary Figure 1

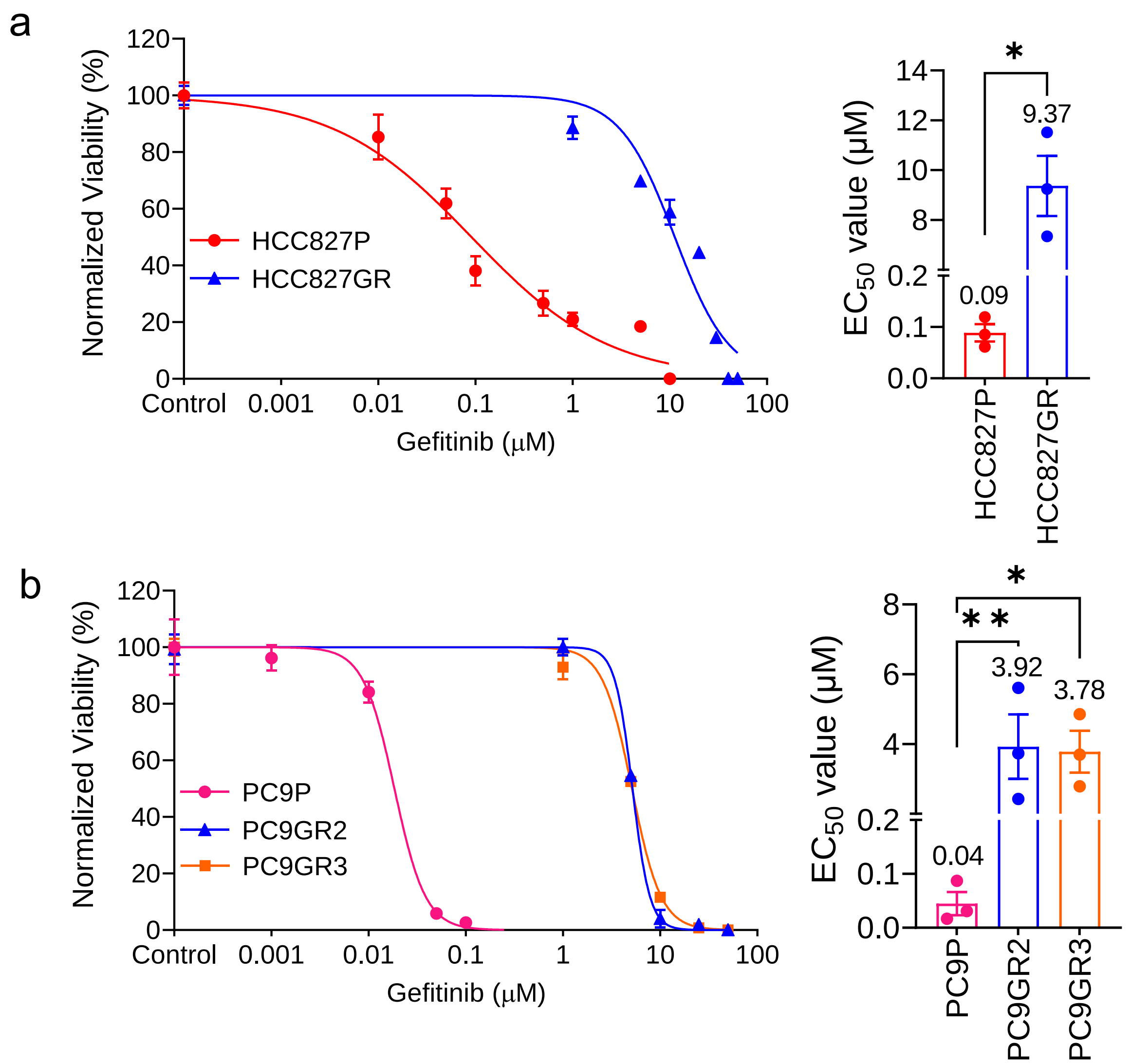

Supplementary Figure 1: Gefitinib-resistant human NSCLC cells exhibit 100-fold increase in EC_{50} values relative to their parental equivalents. **a-b** Cell viability assays using MTS-1 reagent were performed in parental cell lines HCC827P (a) and PC9P (b), as well as gefitinib-resistant HCC827GR (a), PC9GR2 (b), and PC9GR3 (b) cells treated with increasing concentration of gefitinib for 72h. The half maximal effective concentration (EC_{50}) of gefitinib was generated by plotting cell viability results in the normalized dose-response curve available in GraphPad Prism 9 software. The bar graphs on the right-hand side represent average EC_{50} values of three independent experiments. Numerical values illustrate the mean and error bars denote SEM. *$P \leq 0.05$ and **$P \leq 0.01$, 2-way unpaired t-test (a) and 1-way ANOVA followed by Dunnett's multiple comparison testing (b).

Supplementary Figure 2

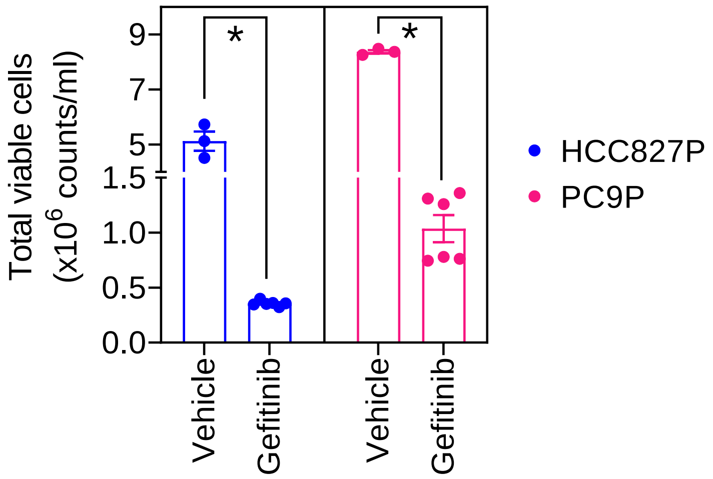

Supplementary Figure 2: Gefitinib-mediated cell death. Human EGFR-mutated NSCLC cells, HCC827 and PC9, were treated with 10 nM gefitinib for six days. Trypan blue exclusion analysis was performed to determine viable cells following 6 days of gefitinib treatment. Each circle depicted on the bar graph represents an independent biological replicate. Data are presented as mean ± SEM. **P*≤0.05, 2-way unpaired t-test.

Supplementary Figure 3

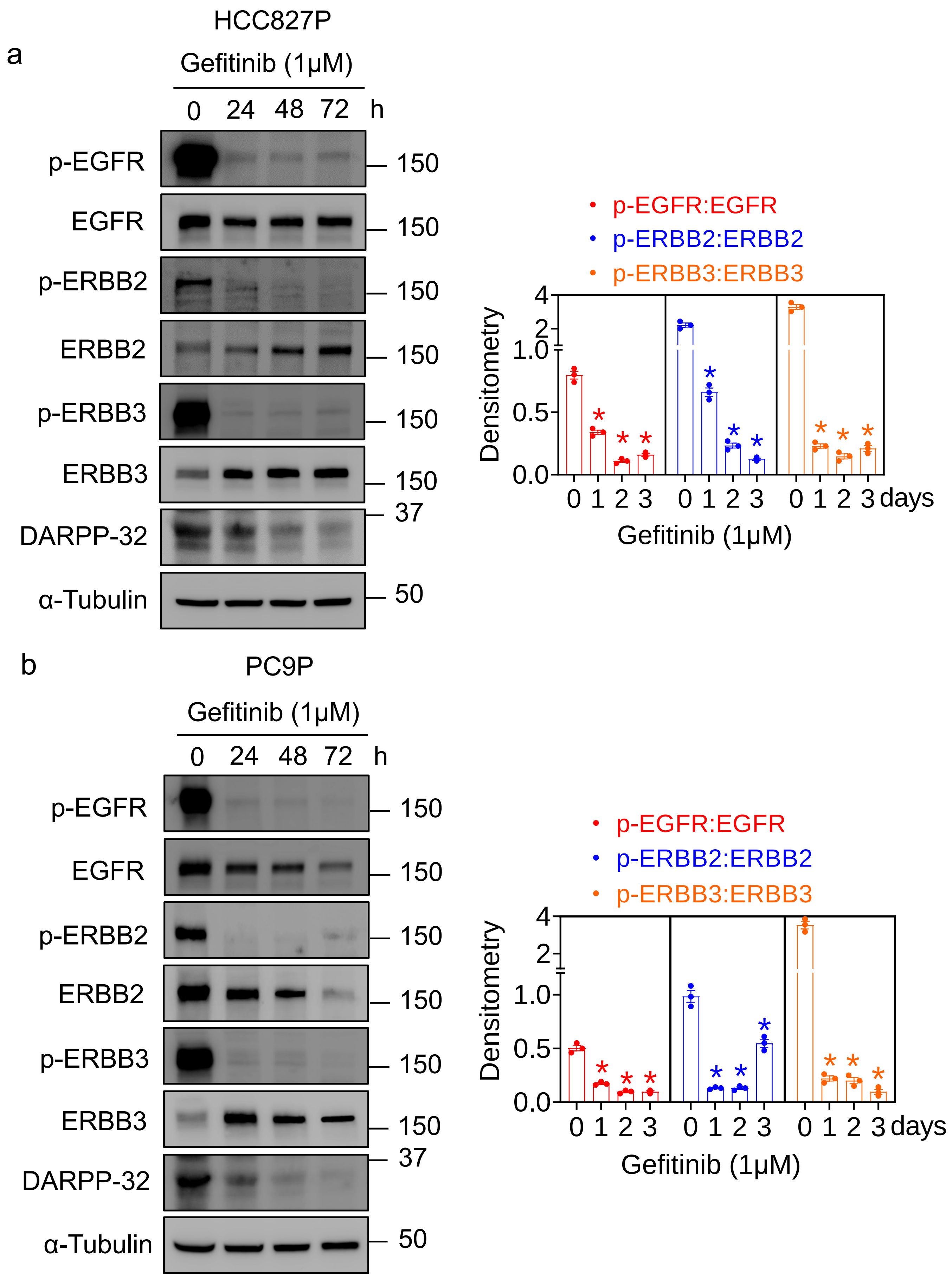

Supplementary Figure 3: Gefitinib blocks EGFR phosphorylation in EGFR-mutated human NSCLC cells. **a-b** HCC827P (a) and PC9P (b) cells treated with 1 µM gefitinib for indicated times were lysed in RIPA buffer and phosphorylated EGFR (p-EGFR), total EGFR (EGFR), phosphorylated ERBB2 (p-ERBB2), total ERBB2 (ERBB2), phosphorylated ERBB3 (p-ERBB3), total ERBB3 (ERBB3), DARPP-32, and α-tubulin (loading control) proteins were detected by immunoblotting of cell lysates. Quantification of p-EGFR, p-ERBB2, and p-ERBB3 proteins was performed using Image J software. The ratio of each phosphorylated protein relative to its unphosphorylated equivalent (i.e., total protein) have been represented in the bar graph (right side). Each circle on the bar graph represents an independent experiment. Mean ± SEM, n=3. **P*≤0.05, 1-way ANOVA followed by Dunnett's multiple comparison testing.

Supplementary Figure 4

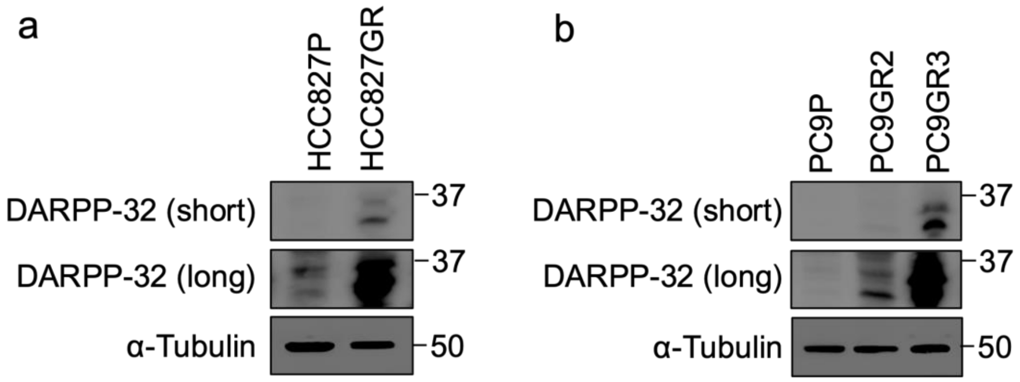

Supplementary Figure 4: DARPP-32 is upregulated in gefitinib-resistant cell lines. **a** Human NSCLC cell lines, HCC827P and HCC827GR, were lysed and immunoblotted to detect DARPP-32 and α-tubulin (loading control) protein expression. **b** PC9P, PC9GR2, and PC9GR3 cell lysates were separated by SDS-PAGE and immunoblotting was performed using antibodies against DARPP-32 and α-tubulin. **a, b** Both “short” and “long” immunoblotting development exposures are depicted.

Supplementary Figure 5

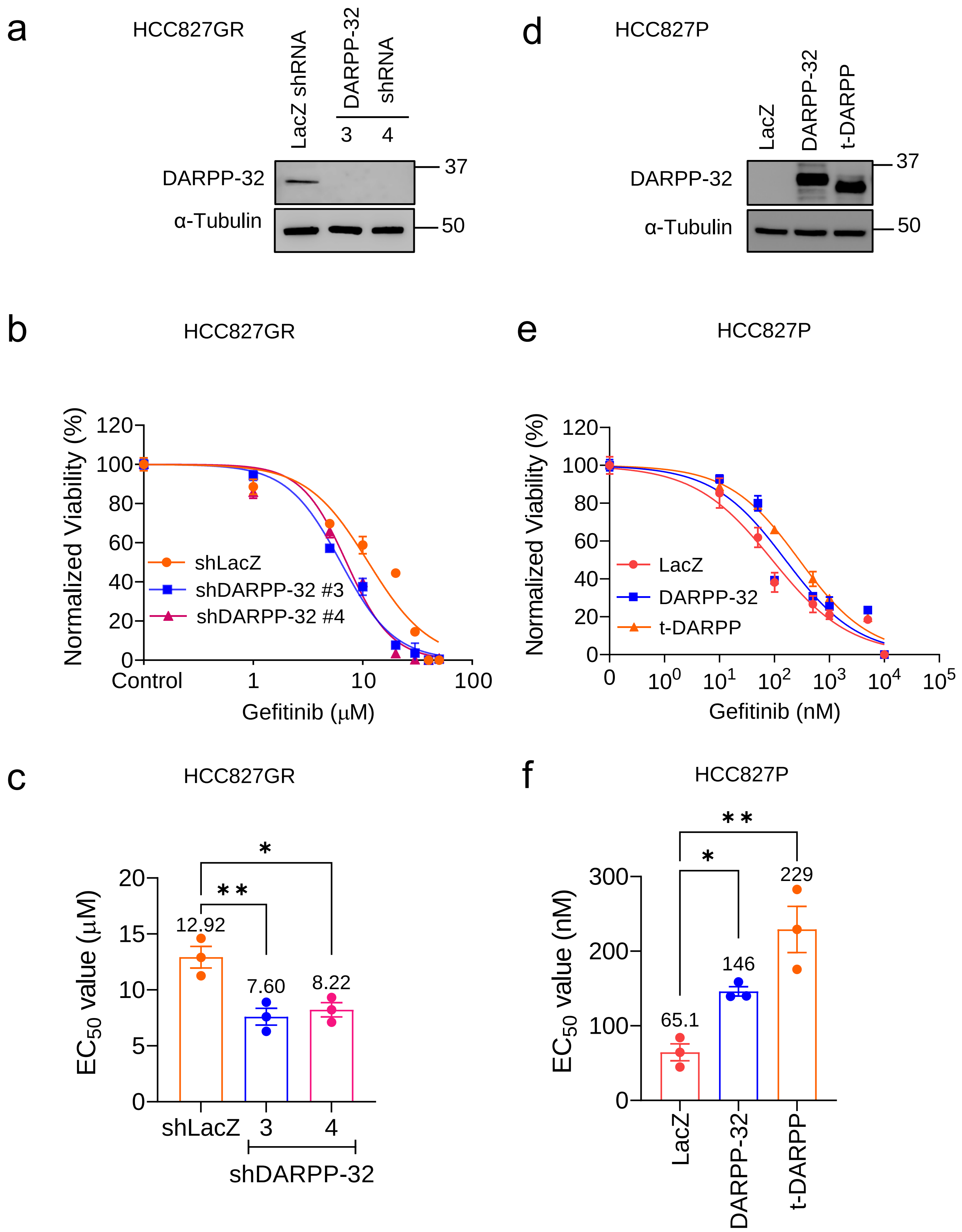

Supplementary Figure 5: DARPP-32 overexpression increases NSCLC cell survival. **a** Human LUAD HCC827GR cells transduced with lentivirus encoding control (LacZ) or DARPP-32 shRNAs were

immunoblotted with DARPP-32 and α-tubulin (loading control) antibodies. **b** HCC827GR cells were transduced with control (LacZ) or DARPP-32 shRNAs and seeded into 96-well cell culture plates. Cells were treated with increasing concentrations of gefitinib, and colorimeter-based cell survival assays were conducted using MTS1 reagents. **c** The half maximal effective concentration (EC_{50}) of gefitinib was determined from MTS1 survival assays and plotted. **d** HCC827P cells were transduced with retrovirus encoding control (LacZ), DARPP-32 or t-DARPP overexpressing clones. Cells were lysed and immunoblotting was performed to detect DARPP-32 isoforms and α-tubulin (control). **e** Cell survival assays were performed using HCC827P cells stably overexpressing LacZ, DARPP-32 or t-DARPP proteins exposed to increasing concentrations of gefitinib. **f** Gefitinib-treated HCC827P cells overexpressing LacZ or DARPP-32 isoforms were subjected to MTS1-based cell survival assays and EC_{50} of gefitinib was calculated. Each open circle on a graph represents an independent experiment. All bar graphs represent mean ± SEM (n=3). *P<0.05, **P<0.01, and ***P<0.001, one-way ANOVA followed by Dunnett’s test for multiple comparison.

Supplementary Figure 6

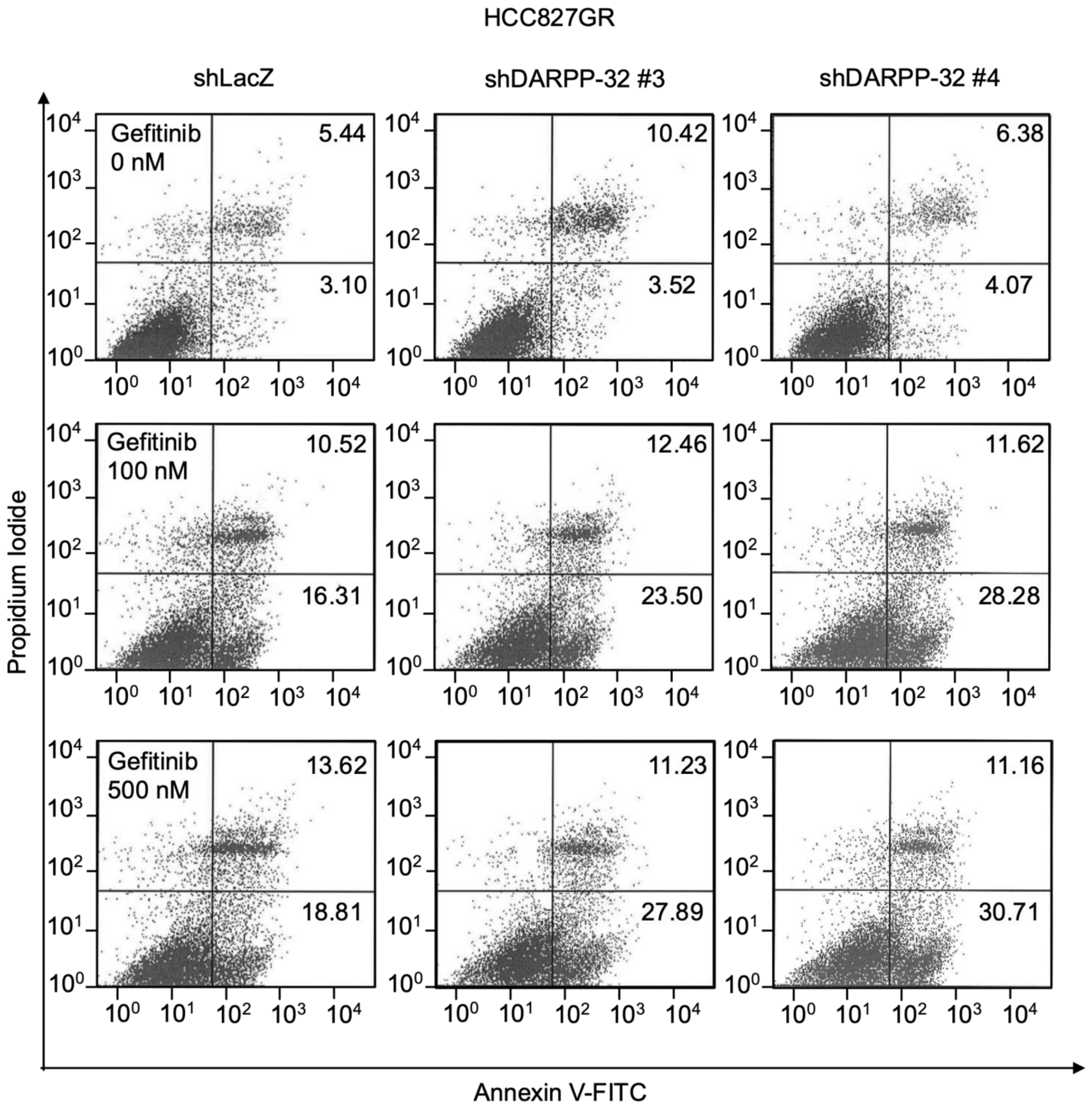

Supplementary Figure 6: DARPP-32 ablation increases cell apoptosis in the presence of gefitinib. Human NSCLC HCC827GR cells transduced with control (LacZ) or DARPP-32 shRNAs were incubated with anti-annexin V antibodies conjugated with FITC followed by propidium iodide incorporation. The total number of annexin V-positive cells was determined using flow cytometry-based apoptosis assays. The numerical values shown on quadrants of the scatter plots represent the percentage of total cells in one single representative experiment.

Supplementary Figure 7

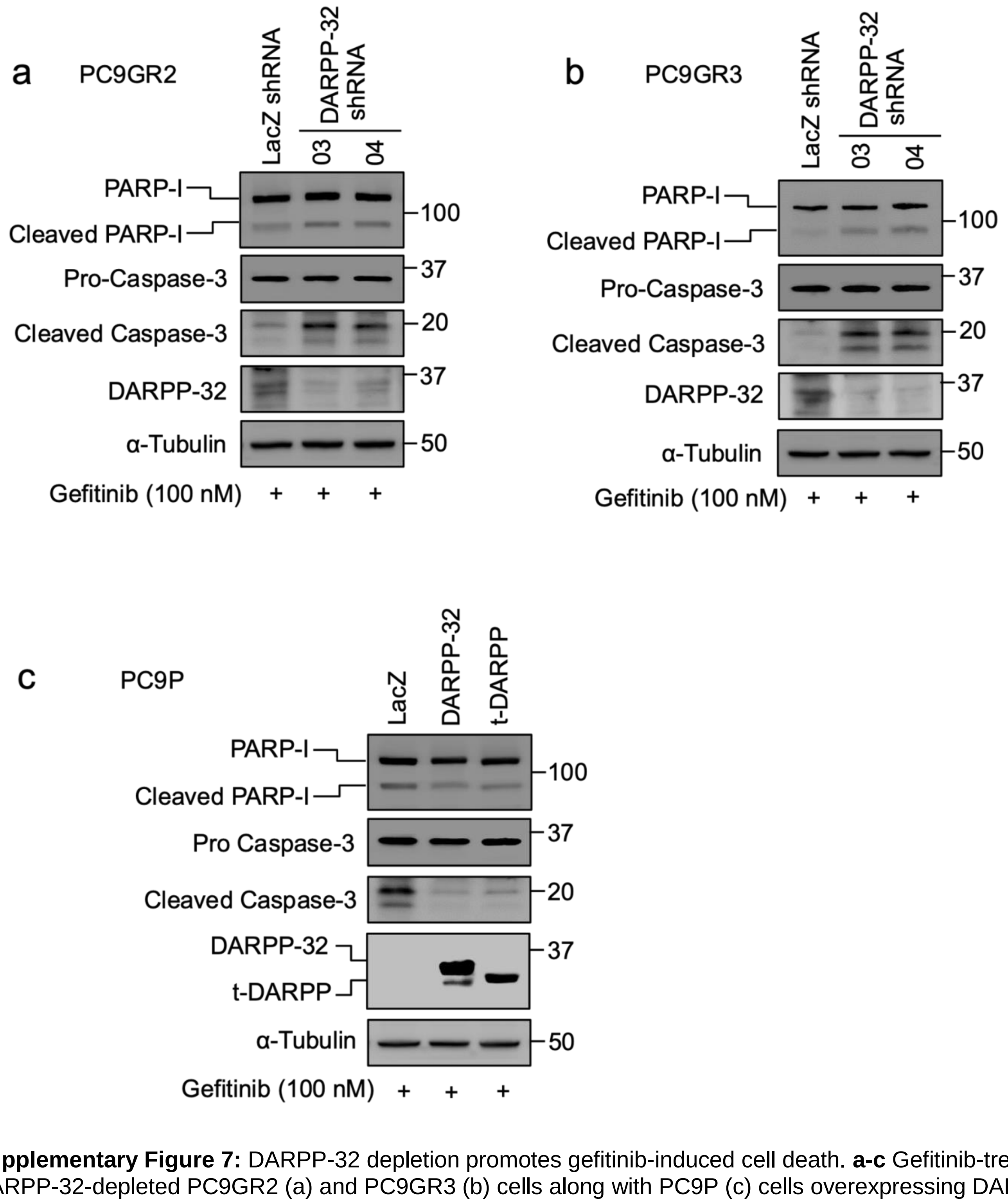

Supplementary Figure 7: DARPP-32 depletion promotes gefitinib-induced cell death. **a-c** Gefitinib-treated DARPP-32-depleted PC9GR2 (a) and PC9GR3 (b) cells along with PC9P (c) cells overexpressing DARPP-32 isoforms were lysed and western blotting was performed using antibodies against cleaved and uncleaved PARP-I, cleaved and uncleaved (i.e., pro-) caspase-3, DARPP-32 and α-tubulin (loading control).

Supplementary Figure 8

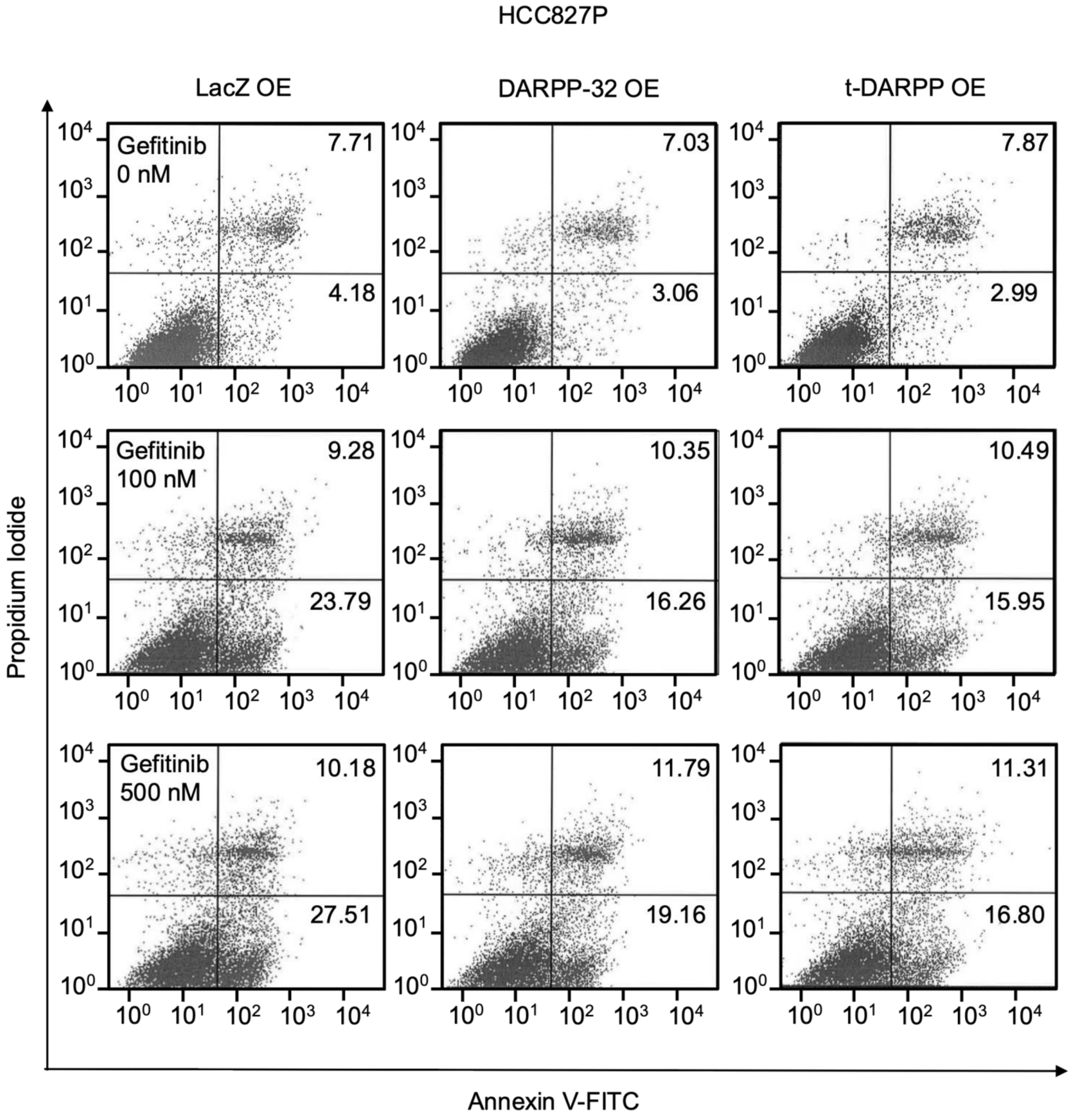

Supplementary Figure 8: DARPP-32 overexpression suppresses cellular apoptosis upon gefitinib treatment. HCC827P cells were transduced with retrovirus containing control (LacZ)-, DARPP-32- or t-DARPP-overexpressing clones. Flow cytometry-based apoptosis assays were performed in gefitinib-treated cells using FITC-conjugated anti-annexin V antibodies along with propidium iodide. The numerical values shown on quadrants of the scatter plots represent the percentage of total cells in one single representative experiment.

Supplementary Figure 9

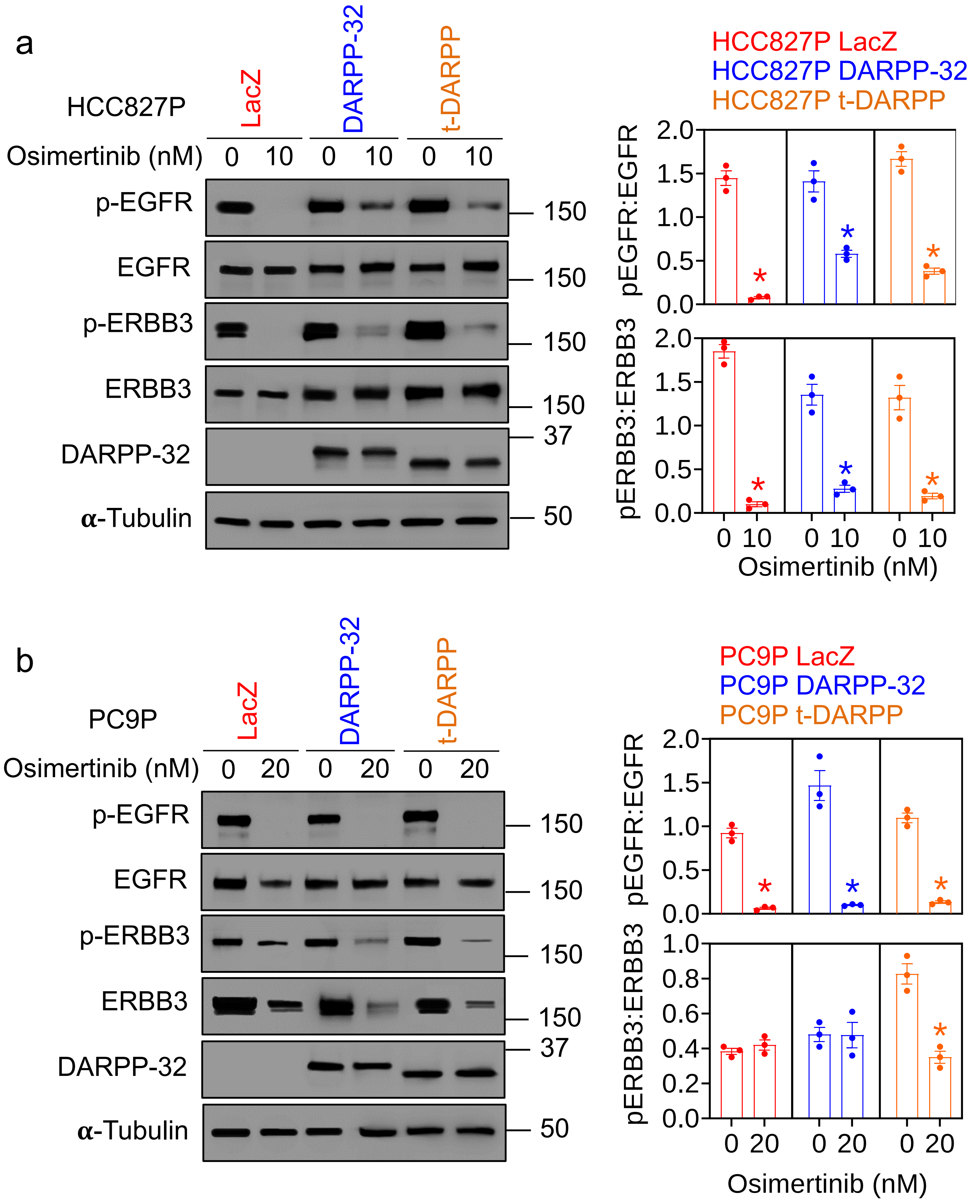

Supplementary Figure 9: Osimertinib reduces p-EGFR and p-ERBB3 expression in EGFR-mutated LUAD cells overexpressing DARPP-32 isoforms. **a-b** HCC827P (a) and PC9P (b) cells transduced with retrovirus encoding LacZ, DARPP-32, or t-DARPP were treated with the indicated doses of third-generation EGFR TKI, osimertinib, for 24h. Cells were then lysed using 1X RIPA buffer and immunoblotted using primary antibodies

against p-EGFR, EGFR, p-ERBB3, ERBB3, DARPP-32, and α-tubulin (loading control). Quantification of the immunoblot band intensities was performed using Image J software and has been presented as an average of three independent experiments. Bar graphs represent mean ± SEM. *$P \leq 0.05$, 2-way unpaired t-test.

Supplementary Figure 10

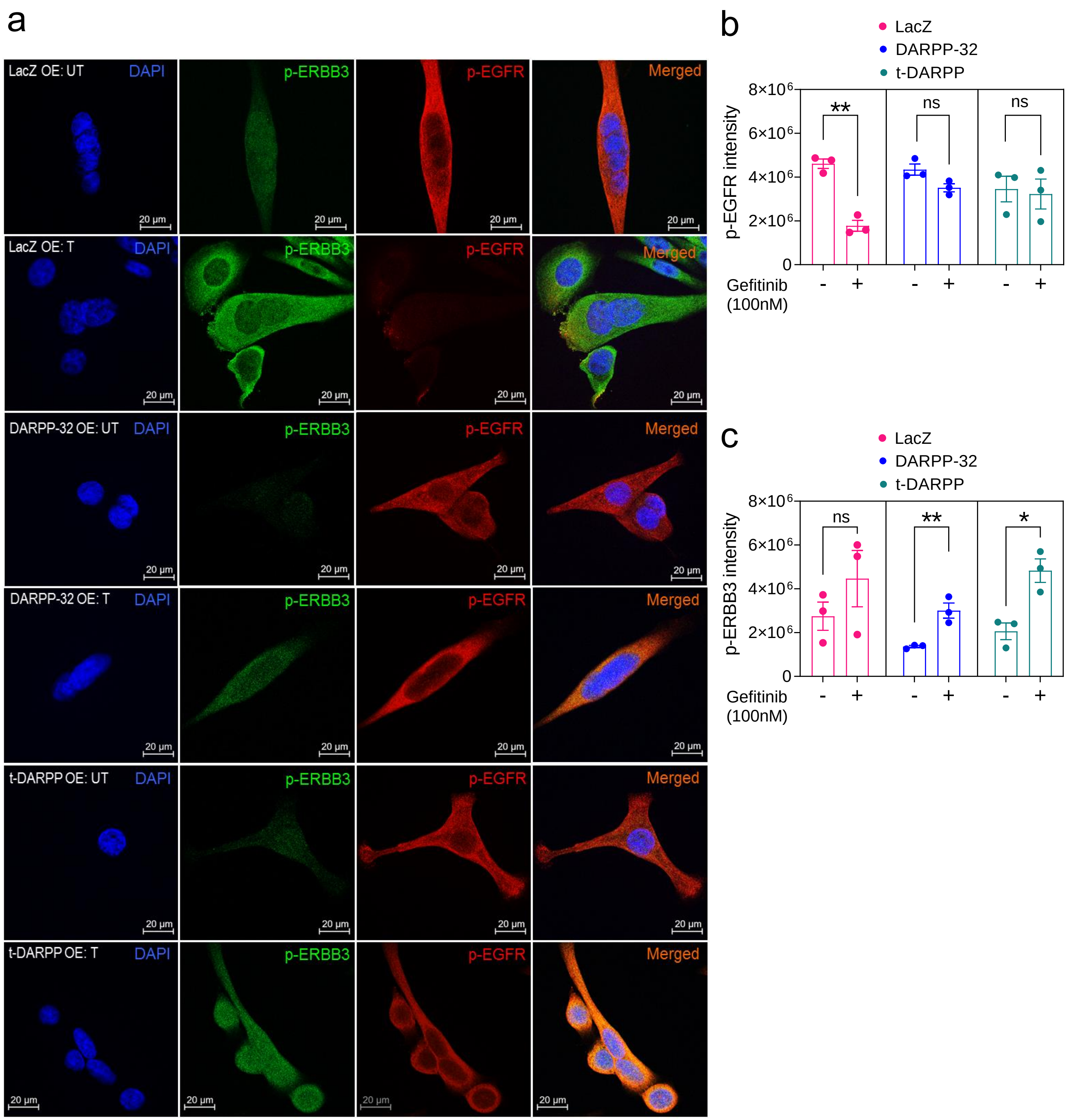

Supplementary Figure 10: Overexpression of DARPP-32 isoforms increases p-ERBB3 expression. **a** Human NSCLC PC9P cells transduced with retrovirus containing control (LacZ)-, DARPP-32- or t-DARPP-overexpressing clones were treated with vehicle (UT) or 100 nM gefitinib (T) and immunofluorescence experiments were performed using primary antibodies against p-ERBB3 (green) and p-EGFR (red). Nuclei were stained with DAPI (blue). **b-c** Average red (b) and green (c) fluorescence intensity of 6-10 random microscopic fields for each sample has been reported. Experiments were repeated at least three times. Scale bar, 20 µm. Bar graphs indicate mean ± SEM (n=3). *P<0.05 and **P<0.01, 2-way unpaired t-test.

Supplementary Figure 11

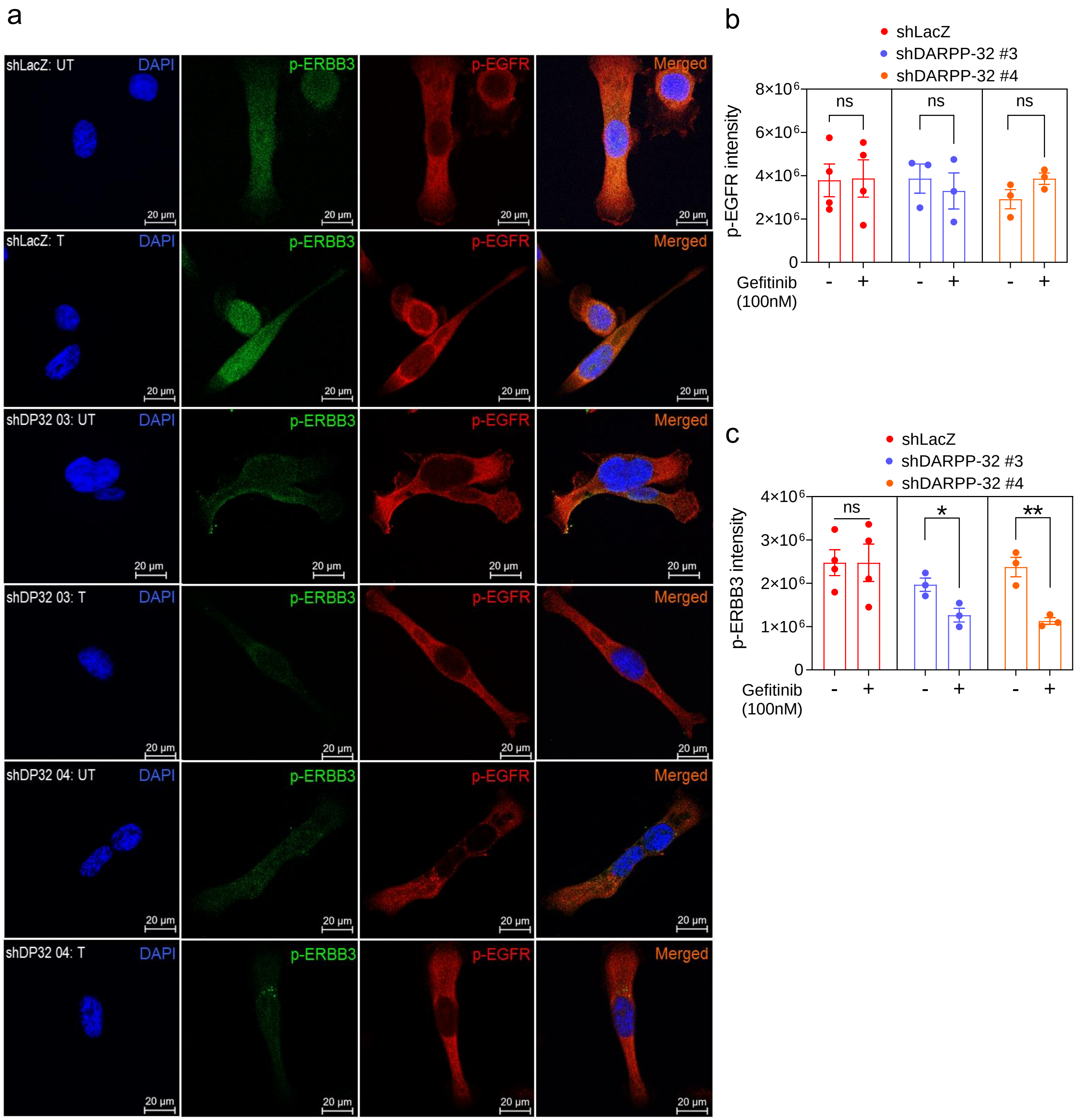

Supplementary Figure 11: Expression of p-ERBB3 is controlled by DARPP-32. **a** PC9GR3 cells were transduced with lentivirus containing control (LacZ) or DARPP-32 shRNAs. Cells treated with vehicle (UT) or 100nM gefitinib (T) were fixed, permeabilized, and incubated with primary antibodies that detect p-ERBB3 (green) and p-EGFR (red) proteins. DAPI-stained nuclei were represented in blue color. **b-c** Expression of p-EGFR (b) and p-ERBB3 (c) was reported by calculating average fluorescence intensity of 6-10 random microscopic fields for each sample. Each circle on a graph represents an independent experiment. Scale bar, 20 μm. Results represent mean ± SEM (n=3). *P<0.05 and **P<0.01, 2-way unpaired t-test.

Supplementary Figure 12

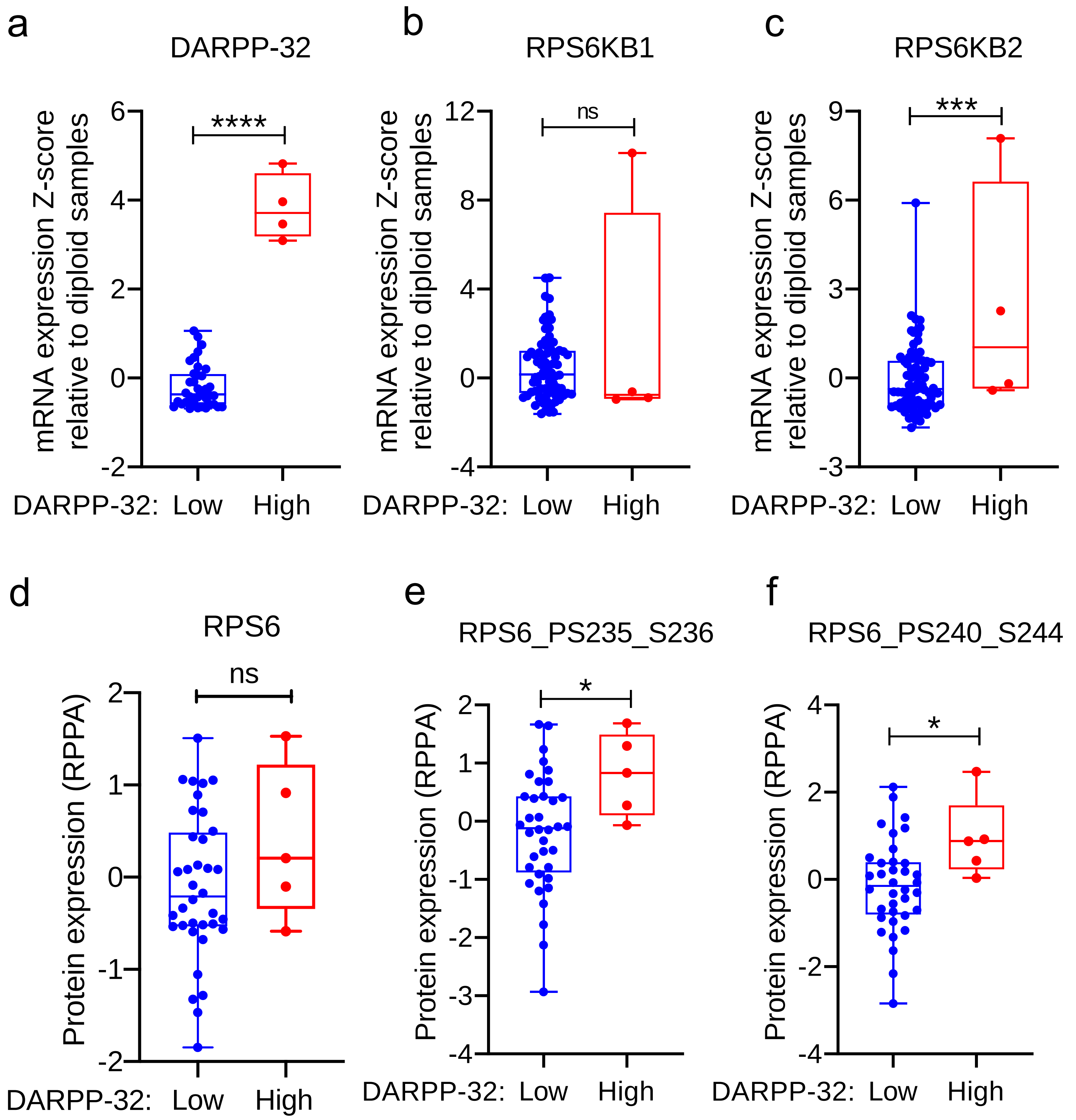

Supplementary Figure 12: Integrative approach to analyze ribosomal protein S6 (RPS6) expression in human LUAD patients using cBioPortal. **a-c** Box plot showing the relative mRNA expression of DARPP-32 (a), RPS6KB1 (b), and RPS6KB2 (c) in 80 human LUAD patient samples from The Cancer Genome Atlas (TCGA) study. **d-f** Box plots representing the relative amount of total- (d) and phospho-RPS6 (e-f) proteins by RPPA in DARPP-32-altered human EGFR-mutated LUAD patient-derived specimens. Based on the DARPP-32 expression, patients were divided between DARPP-32 -low (n=76) vs -high (n=4) groups. Each dot on box plots represents a single patient. *P<0.05, ***P<0.001, and ****P<0.0001, 2-way unpaired t-test.

Supplementary Figure 13

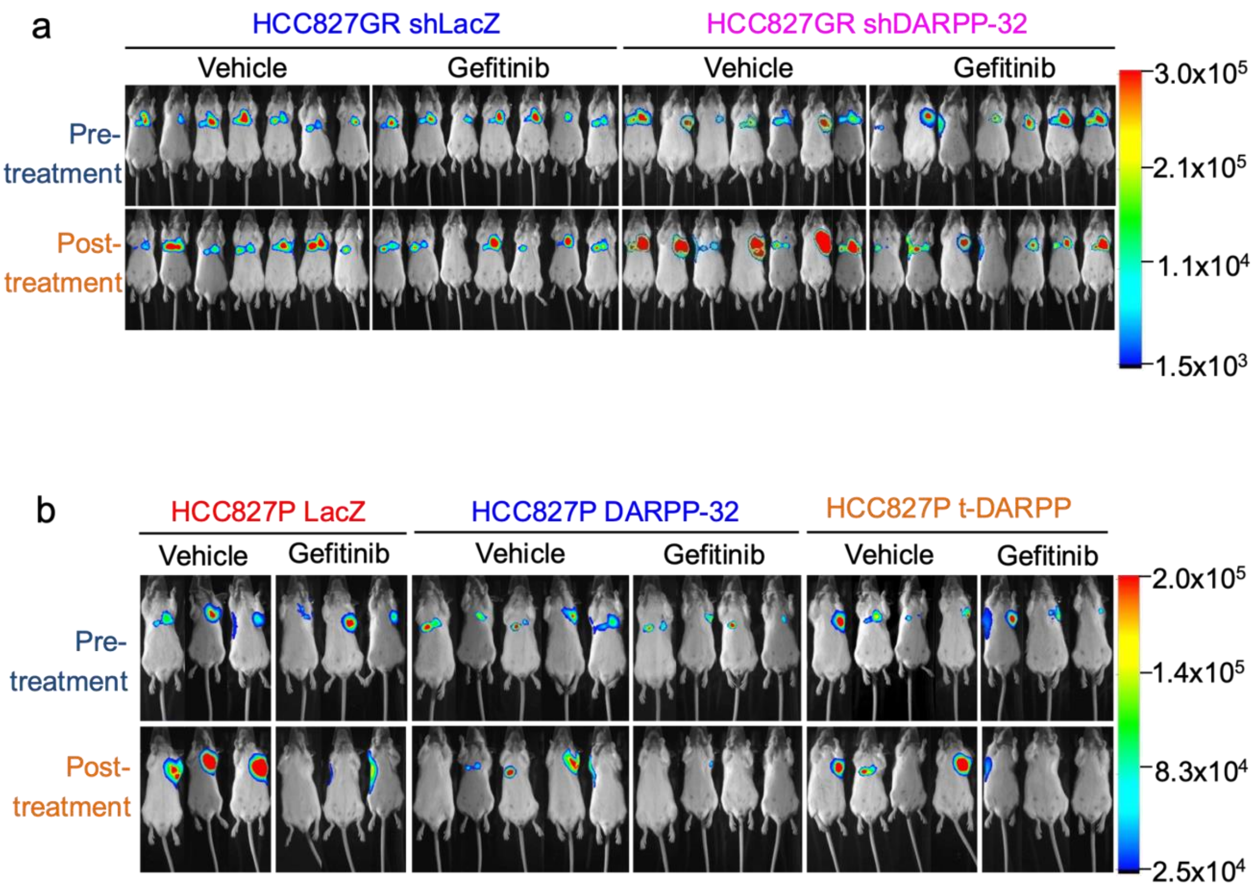

Supplementary Figure 13: Pre- and post-treatment luminescence images of vehicle- and gefitinib-treated mice. **a** DARPP-32-depleted luciferase-labeled human HCC827GR cells were orthotopically injected into the left thoracic cavity of SCID mice. Mice administered either vehicle or gefitinib (25 mg/kg) were imaged for luminescence before and after treatment. **b** Luciferase-labeled human HCC827P cells transduced with retrovirus encoding control (LacZ), DARPP-32 or t-DARPP cDNAs were orthotopically injected into the left thoracic cavity of SCID mice. After establishment of the tumor, mice were treated with vehicle or gefitinib (25 mg/kg) three times in a week. Luminescence images of mice were taken pre- and post-treatment. The colored bar represents the numerical value of luminescence.

Supplementary Figure 14

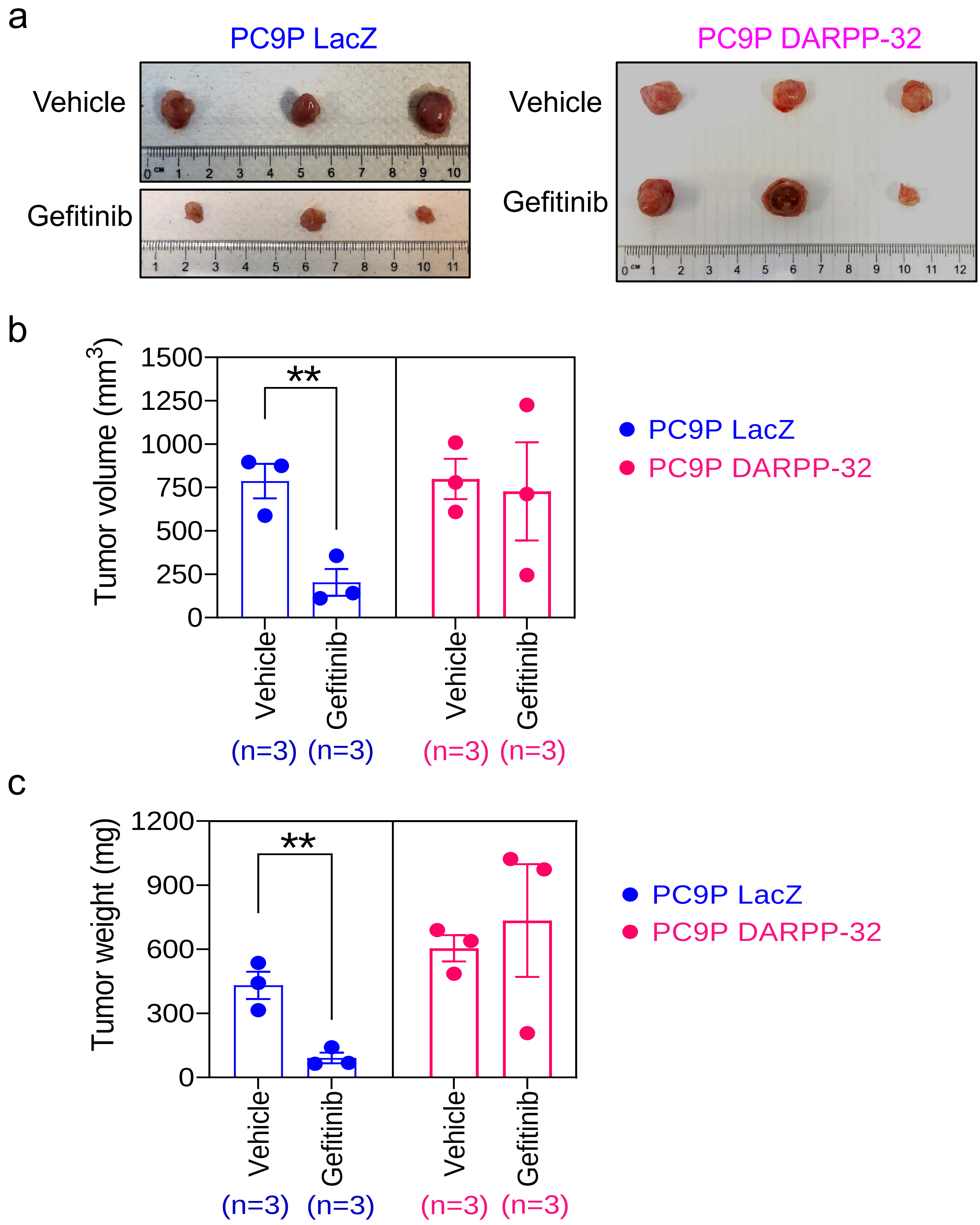

Supplementary Figure 14: Overexpression of DARPP-32 suppresses gefitinib-mediated tumor reduction *in vivo*. **a-c** SCID mice were subcutaneously injected with PC9P cells stably overexpressing control (LacZ) or DARPP-32 cDNAs and treated with vehicle or gefitinib (25 mg/kg). At the experimental endpoint, mice were sacrificed and xenografted tumors were extirpated. Photographs of extirpated tumors were taken to visualize gross morphology (a). Tumor volume was calculated from caliper-based measurements following extirpation (b). Extirpated tumors were weighed using a digital balance (c). Each open circle depicted on the bar graphs represents an individual mouse. Bar diagrams show mean ± SEM. **P<0.01, 2-way unpaired t-test.

Supplementary Figure 15

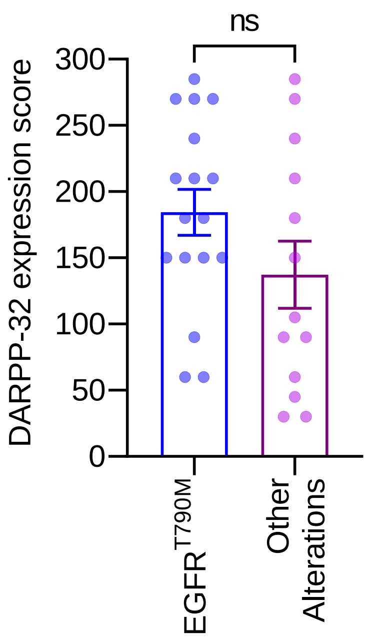

Supplementary Figure 15: DARPP-32 protein expression in specimens derived from EGFR-mutated LUAD patients upon first generation EGFR TKI refractory disease progression. Tumor specimens from LUAD patients with EGFR TKI-refractory LUAD harboring either EGFR T790M mutations (n=17) or other genetic alterations (n=13 total, including either unknown alterations (n=9), MET amplifications (n=2), KRAS G12D mutation (n=1), or MYC amplification and TP53 mutation (n=1)) were immunostained using primary antibodies against DARPP-32. Expression score of DARPP-32 calculated by multiplying the staining intensity score (0-3) by the percent of positive tumor cells was plotted in the bar graph. Each circle shown on the graph represents single patient. *P<0.05, 2-way unpaired t-test.

Supplementary Table 1

ID	Stage	Age	Sex	Pathology	Genotype	Baseline Pathology Evaluation					Resistance Pathology Evaluation					Treatment	Response	Tumor shrank	Resistant Mechanism	PFS
						DARPP-32	EGFR	p-EGFR	ERBB3	p-ERBB3	DARPP-32	EGFR	p-EGFR	ERBB3	p-ERBB3					
1	IV	41	1	ADC	L858R	60	90	0	0	0	270	300	100	100	20	gefitinib	PR	-70%	EGFR T790M	19
2	IV	56	2	ADC	L858R	210	120	5	0	10	270	210	50	90	40	erlotinib	PR	-65%	EGFR T790M	11
3	IV	48	2	ADC	L858R	0	150	60	0	0	210	150	100	100	10	erlotinib	PR	-50%	EGFR T790M	10
4	IV	48	2	ADC	L858R	0	30	0	0	0	150	140	7	100	15	gefitinib	PR	-40%	EGFR T790M	10
5	IV	53	2	ADC	L858R	240	0	0	0	0	285	120	15	5	15	gefitinib	PR	-40%	EGFR T790M	13
6	IV	62	2	ADC	L858R	60	60	0	0	0	90	150	0	0	0	gefitinib	SD	-12.50%	KRAS exon 2 G12D	6
7	IV	70	1	ADC	L858R	60	0	0	0	0	285	40	15	15	0	gefitinib	PR	-60%	MYC amplification and TP53 mutation	15
8	IV	62	2	ADC	L858R	180	50	0	0	0	210	0	0	0	0	gefitinib	PR	-30%	Unknown	21
9	IV	61	1	ADC	L858R	150	150	0	0	0	105	150	0	35	0	gefitinib	PR	-70%	Unknown	14
10	IV	54	2	ADC	L858R	240	50	0	0	0	210	210	0	0	0	erlotinib	PR	-30%	EGFR T790M	30
11	IV	51	2	ADC	L858R	60	0	0	0	0	180	80	10	30	0	gefitinib	SD	-10%	EGFR T790M	25
12	IV	66	2	ADC	L858R	0	210	0	0	0	30	150	20	10	30	gefitinib	PR	-60%	MET Amplification	8
13	IV	55	2	ADC	L858R	240	100	0	10	5	240	270	0	60	10	erlotinib	PR	-30%	EGFR T790M	8
14	IV	66	2	ADC	L858R	90	120	0	0	0	150	270	20	0	0	erlotinib	PR	-80%	MET Amplification	11
15	IV	47	2	ADC	L858R	0	0	0	0	0	240	100	20	0	0	gefitinib	SD	-15%	Unknown	3
16	IV	66	2	ADC	L858R	180	0	0	0	0	150	180	30	15	0	erlotinib	PR	-30%	EGFR T790M	21
17	IV	63	1	ADC	L858R	120	120	40	0	0	180	180	10	10	0	gefitinib	PR	-35%	EGFR T790M	9
18	IV	62	1	ADC	L858R	180	30	0	0	0	150	210	10	100	10	erlotinib	PR	-35%	EGFR T790M	24
19	IV	70	1	ADC	L858R	100	30	20	0	0	90	150	70	50	0	gefitinib	PR	-30%	EGFR T790M	22
20	IV	62	1	ADC	L858R	210	30	0	0	0	30	30	50	10	0	gefitinib	PR	-30%	Unknown	4
21	IV	81	1	ADC	L858R	240	80	0	0	0	60	120	10	0	30	erlotinib	PR	-50%	EGFR T790M, EGFR amplification	12
22	IV	61	1	ADC	L858R	100	180	0	10	15	60	270	50	10	10	erlotinib	SD	-15%	EGFR T790M	26
23	IV	54	2	ADC	L858R	240	80	0	0	0	180	100	0	60	0	gefitinib	PR	-50%	Unknown	15
24	IV	48	2	ADC	L858R	20	160	0	0	0	60	210	60	60	5	erlotinib	SD	-20%	Unknown	3
25	IV	66	1	ADC	L858R	20	270	30	0	0	90	150	0	30	0	gefitinib	PR	-70%	Unknown	18
26	IV	55	2	ADC	L858R	240	60	0	5	5	210	210	0	15	25	gefitinib	PR	-35%	EGFR T790M	13
27	IV	66	1	ADC	L858R	240	50	0	5	5	270	100	10	10	0	erlotinib	PR	-30%	Unknown	12
28	IV	59	2	ADC	L858R	40	300	0	0	0	45	210	25	40	40	gefitinib	PR	-35%	Unknown	21
29	IV	62	1	ADC	L858R	40	80	0	10	0	150	120	0	0	20	erlotinib	PR	-42%	EGFR T790M, EGFR amplification	7.8
30	IV	70	1	ADC	L858R	60	40	0	0	0	270	160	0	0	0	erlotinib	SD	-20%	EGFR T790M	19
