# Supplementary material for: DARPP-32 promotes ERBB3-mediated resistance to molecular targeted therapy in EGFR-mutated lung adenocarcinoma
Source: Oncogene. 2021 Oct 21;41(1):83–98. doi: 10.1038/s41388-021-02028-5 (PMC8529229; doi:10.1038/s41388-021-02028-5)
Supplement: Supplementary file 2 — Supplementary methods. [file 41388_2021_2028_MOESM2_ESM.pdf]

SUPPLEMENTARY METHODS:

Generation of stable cell lines: Human DARPP-32 and t-DARPP cDNAs cloned in pcDNA3.1 were kindly provided by Dr. Wael El-Rifai at University of Miami[1]. To generate retrovirus, we first subcloned FLAG-tagged DARPP-32 and t-DARPP cDNAs into pMMP vectors that were a kind gift from Dr. Debabrata Mukhopadhyay at Mayo Clinic in Jacksonville, Florida[2]. HEK-293T cells were next transfected with pMMP vectors together with retrovirus packaging plasmids using Effectene transfection reagent (Qiagen) according to the manufacturer's protocol. Two days after transfection, retrovirus was collected from the cell culture medium, concentrated using Retro-X concentrator (Takara), and used immediately to transduce human NSCLC cell lines, HCC827P and PC9P, as described previously[3].

To prepare lentivirus, LacZ shRNA (control) and 4 different DARPP-32 shRNAs cloned in pLKO.1 plasmids (Sigma) were transfected in human HEK-293T cells along with their corresponding packaging plasmids. Lentivirus was isolated from cell culture medium 48h after transfection, concentrated using Lenti-X concentrator (Takara), and used immediately to transduce HCC827GR, PC9GR2, and PC9GR3 lung cancer cell lines, as reported previously[3]. Stable DARPP-32 knockdown cells were used for experiments following 72h of puromycin (Sigma) selection.

Xenografts of HCC827P and HCC827GR cells transduced with lentivirus encoding the luciferase gene were used to determine tumor growth in orthotopic murine models. Briefly, luciferase gene encoding lentivirus was prepared by transfecting MSCV Luciferase PGK-hygro plasmids obtained through Dr. Scott Lowe via Addgene (#18782) along with their corresponding packaging plasmids into HEK-293T cells. Two days post-transfection, lentivirus collected from the cell culture media was concentrated using Retro-X concentrator (Takara). The concentrated lentivirus was used immediately to transduce human NSCLC cell lines, HCC827P and HCC827GR, as described previously[3]. Luciferase-labeled stable human NSCLC cells were obtained following 72h of hygromycin (Sigma) selection after transduction.

Antibodies: For detection of proteins in immunoblotting experiments, we purchased monoclonal antibodies (1 µg/µl) from Cell Signaling Technology against phosphorylated EGFR (Y1068; Cat no.: 3777; Dilution 1:1000), total EGFR (Cat no.: 4267, Dilution 1:1000), phosphorylated ERBB2 (Y1221/1222; Cat no.: 2243; Dilution 1:1000), total ERBB2 (Cat no.: 4290, Dilution 1:1000), phosphorylated ERBB3 (Y1289; Cat no.: 2842; Dilution 1:1000), total ERBB3 (Cat no.: 12708, Dilution 1:1000), PARP-I (Cat no.: 9542; Dilution 1:1000), Caspase-3 (Cat no.: 9662; Dilution 1:1000), Cleaved Caspase-3 (Cat no.: 9664; Dilution 1:1000), phosphorylated AKT (S473; Cat no.: 4060; Dilution 1:1000), total AKT (Cat no.: 4691; Dilution 1:1000), phosphorylated ERK1/2 (T202/Y204; Cat no.: 4370; Dilution 1:1000), total ERK1/2 (Cat no.: 4695; Dilution 1:1000). Monoclonal antibodies (200 µg/ml) to detect DARPP-32 (Cat no.: sc-135877; Dilution 1:200) and α-Tubulin (Cat no.: sc-5286; Dilution 1:1000) protein were obtained from Santa Cruz Biotechnology. Horseradish peroxidase (HRP)-conjugated secondary antibodies (1 µg/µl) purchased from Cell Signaling Technology were used to detect primary antibodies raised in either rabbit (Cat no.: 7074; Dilution 1:5000) or mouse (Cat no.: 7076; Dilution 1:5000).

Immunoblotting: Radioimmunoprecipitation assay (RIPA) buffers (Millipore) containing protease (Roche) and phosphatase inhibitors (Millipore) were used to lyse human NSCLC cell lines. Proteins quantified using the Quick Start Bradford protein assay reagents (Bio-Rad) were separated via 4-20% gradient SDS-PAGE (Bio-Rad) and transferred to polyvinyl difluoride membranes (PVDF; Millipore). Membranes blocked with 5% bovine serum albumin (BSA; Sigma-Aldrich) were then incubated with primary and corresponding secondary antibodies overnight and for 2h, respectively. Enzyme-based chemiluminescence substrate (Thermo Fisher Scientific) was used to detect antibody-reactive protein bands.

Cell survival assay: Human LUAD cell lines, HCC827P and HCC827GR, each plated in a 96-well microplate at a concentration of 5000 cells/well, were used to determine cell survival in the presence of increasing concentrations of gefitinib. Seventy-two hours post-gefitinib treatment, cell viability was assessed using MTS1-based CellTiter 96® AQueous One System (Promega). Absorbance of cell culture medium recorded at 490 nm using a Synergy Neo2 microplate spectrophotometer (Biotek) was used to calculate EC_{50} values of gefitinib in different experimental groups.

Human LUAD parental cells, HCC827P and PC9P, each plated at a density of 100,000 cells per well in a 6-well cell culture plate were treated with 10 nM of gefitinib for 6 days. At the end of drug treatment, cells were washed with PBS, trypsinized, and resuspended in 100 µl complete RPMI medium. To determine viable cell numbers, equal volume (i.e., 100 µl) of trypan blue dye was added to the cell suspension. Viable cells rejecting trypan blue dye were counted using a Cell Drop FL automated cell counter (DeNovix Inc.).

Apoptosis analysis: To determine gefitinib-induced cell apoptosis, $1x10^5$ human EGFR-mutated NSCLC cells plated in 60-mm dishes were stained with fluorescein isothiocyanate (FITC)-conjugated anti-Annexin V antibodies (BD Biosciences) together with propidium iodide (BD Biosciences) following 24h gefitinib treatment. The number of early apoptotic cells (Annexin-positive and propidium-iodide-negative) determined by flow cytometry-based analysis was counted to measure apoptotic cell death.

Immunofluorescence: PC9P and PC9GR3 cells fixed in 4% paraformaldehyde (Boston Bioproducts) were permeabilized in cold methanol (Fisher). Permeabilized cells were then used for immunofluorescence staining using primary antibodies against phosphorylated-EGFR (Y845; BD Biosciences; Cat No.: 558381; Dilution 1:200) and phosphorylated-ERBB3 (Y1289; Cell Signaling Technology; Cat No.: 2842; Dilution 1:200). Secondary antibody staining was performed by incubation with corresponding Alexa Fluor 488-conjugated anti-rabbit antibody (Molecular Probes; Cat no.: A11008; Dilution 1:400) and Alexa Fluor 594-conjugated anti-mouse antibody (Molecular Probes; Cat no.: A11005; Dilution 1:400). Cell nuclei were stained using 4', 6-diamidino-2-phenylindole, dihydrochloride (DAPI), including ProLong® Gold Antifade Reagent (Cell Signaling Technology). Images captured using a Zeiss Apotome.2 microscope (63X objective, 1.25 NA) were processed using ZEN microscope software (Zeiss). The average fluorescence intensity for green and red signals was calculated using ZEN microscope software and reported.

Immunoprecipitation: RIPA buffers (Millipore) supplemented with protease (Roche) and phosphatase inhibitors (Millipore) were used to lyse human NSCLC cell lines for immunoprecipitation studies. Bradford-based protein assay (Bio-Rad) was used to determine the concentration of harvested cell lysates and 500 µg of protein lysate was loaded into the supplied spin column (Catch and Release Immunoprecipitation Kit; Millipore). Immunoprecipitation using antibodies against FLAG (Cell Signaling Technology; Cat No.: 14793), phosphorylated EGFR (Y1068; Cell Signaling Technology; Cat no.: 3777), phosphorylated ERBB3 (Y1289; Cell Signaling Technology; Cat No.: 2842), ERBB3 (Cell Signaling Technology; Cat No.: 12708), and DARPP-32 (Santa Cruz Biotechnology; Cat No.: sc-135877) was achieved by following manufacturer's protocol (Cat no.:17-500; Millipore).

Proximity ligation assay: Human lung adenocarcinoma PC9P and PC9GR3 cells seeded in chamber slides at a density of $1x10^4$ cells/well were incubated with or without 100nM gefitinib for 24h. Cells were then washed with PBS (Corning), fixed with 4% paraformaldehyde (Boston Bioproducts), and permeabilized in cold methanol (Fisher). Permeabilized cells were incubated with primary antibodies against phosphorylated-EGFR (Y845; BD Biosciences; Cat No.: 558381; Dilution 1:200) and phosphorylated-ERBB3 (Y1289; Cell Signaling Technology; Cat No.: 2842; Dilution 1:200) diluted in SignalStain® Antibody Diluent (Cell Signaling Technology). Proximity ligation assay (PLA) probes designed to bind to corresponding primary antibodies were ligated, amplified, and washed according to the manufacturer's instructions. Untreated lung cancer cells incubated with rabbit (Cell Signaling Technology, Cat No.: 2729) and mouse non-immune IgG (Cell Signaling

Technology, Cat No.: 5415) were used for negative controls. Slides mounted in ProLong® Gold Antifade Reagent with DAPI (Cell Signaling Technology) were imaged using a Zeiss Apotome.2 microscope (20X objective, 0.8 NA) and processed using ZEN microscope software (Zeiss). PLA results were quantified by counting the number of red PLA signals normalized to the total number of DAPI-stained nucleus using Image J software (Version 1.6.0_24; https://imagej.nih.gov/ij). The average number of PLA signals per cells in 6-10 random microscopic fields for each sample was recorded.

Gene expression analyses: Information regarding EGFR mutations and *PPP1R1B* gene expression in NSCLC were obtained from The Cancer Genome Atlas database (TCGA), an open access database that is publicly available at http://www.cbioportal.org[4, 5]. We selected TCGA Pan Cancer cohort[6] as our data source, which contains detailed information about DNA mutations, copy-number changes, mRNA expression, gene fusions, and DNA methylation of 9,125 tumors. Mutation as well as mRNA expression (i.e. RNA-seq) data in 510 LUAD patients were downloaded from cBioPortal website and classified based on the presence of EGFR mutation. We next subdivided EGFR-mutated patient cohort (n=80) between DARPP-32 low (n=76) vs high (n=4) groups based on the *PPP1R1B* mRNA expression. The mRNA expression of PI3K/AKT/mTOR downstream targets (i.e. *RPS6KB1* and *RPS6KB2*) in 80 EGFR-mutated patients were obtained by sorting the RNA-seq data according by unique patient IDs, such as “TCGA-05-4244”. Briefly, RNA-seq results were downloaded from cBioPortal website by entering gene symbols. Downloaded results were then sorted based on the patient ID. Normalized mRNA expression of *RPS6KB1* and *RPS6KB2* (i.e. Z-score) were reported in the box plot graph.

Proteome and phosphoproteome data generated from a reverse phase protein array (RPPA) study of specimens derived from NSCLC patients (n=360) were extracted from the TCGA Pan Cancer study database. Based on the information about EGFR mutation status and *PPP1R1B* mRNA expression, we divided the EGFR-mutated patient cohort (n=41) into DARPP-32 low (Z-score<1.5; n=36) and DARPP-32 high (Z-score≥1.5; n=5) groups. Normalized protein and phosphoprotein expression were calculated and reported in the box plot graph.

SUPPLEMENTARY REFERENCES:

1. Chen, Z. *et al.* Gastric tumour-derived ANGPT2 regulation by DARPP-32 promotes angiogenesis. *Gut* **65**, 925-934 (2016).
2. Zeng, H., Dvorak, H.F. & Mukhopadhyay, D. Vascular permeability factor (VPF)/vascular endothelial growth factor (VEGF) peceptor-1 down-modulates VPF/VEGF receptor-2-mediated endothelial cell proliferation, but not migration, through phosphatidylinositol 3-kinase-dependent pathways. *The Journal of biological chemistry* **276**, 26969-26979 (2001).
3. Alam, S.K. *et al.* ASCL1-regulated DARPP-32 and t-DARPP stimulate small cell lung cancer growth and neuroendocrine tumour cell proliferation. *British journal of cancer* **123**, 819-832 (2020).
4. Cerami, E. *et al.* The cBio cancer genomics portal: an open platform for exploring multidimensional cancer genomics data. *Cancer discovery* **2**, 401-404 (2012).
5. Gao, J. *et al.* Integrative analysis of complex cancer genomics and clinical profiles using the cBioPortal. *Science signaling* **6**, pl1 (2013).
6. Sanchez-Vega, F. *et al.* Oncogenic Signaling Pathways in The Cancer Genome Atlas. *Cell* **173**, 321-337.e310 (2018).
